# Elemental Stability in Mixed Noble and Non-Noble Metal High Entropy Alloy Nanoparticle Electrocatalysts

**DOI:** 10.1021/acs.chemmater.6c00856

**Published:** 2026-05-18

**Authors:** Tatiana Priamushko, Nabojit Kar, Pâmella S. Rodrigues, Nilotpal Kapuria, Sara E. Skrabalak, Serhiy Cherevko

**Affiliations:** † Helmholtz Institute Erlangen-Nürnberg for Renewable Energy (IET-2), 28334 Forschungszentrum Jülich GmbH, Cauerstr. 1, Erlangen 91058, Germany; ‡ Department of Chemistry, 1772 Indiana University Bloomington, Bloomington, Indiana 47405, United States; § São Carlos Institute of Chemistry, University of São Paulo, Av. Trab. Sancarlense 400, São Carlos 13566-590, Brazil

## Abstract

High entropy alloys (HEAs) exhibit unique physical and chemical properties that distinguish them from conventional alloys, which makes them highly attractive for electrocatalysis. Their enhanced corrosion resistance is often attributed to the high entropy of mixing; however, predicting and evaluating the stability of individual elements within HEAs remain challenging. Conventional benchmarking approaches, such as accelerated stress tests and ex situ characterization, provide only indirect insights into degradation pathways and lack time-resolved element-specific information. In this work, we combine a scanning flow cell (SFC) with online inductively coupled plasma mass spectrometry (ICP–MS) to enable real-time quantification of metal dissolution from HEA nanoparticle catalysts during electrochemical measurements. This approach allows simultaneous electrochemical characterization and detection of trace metal dissolution at the picogram-*per*-square-centimeter level. The stability of a representative noble-non-noble PdCuPtNiCo HEA is systematically compared with that of bimetallic Pt-based alloys (PtPd, PtNi, and PtCo) in both acidic and alkaline electrolytes. All alloys exhibit high stability in the potential region for the oxygen reduction reaction (ORR), irrespective of pH. However, at higher anodic potentials in acidic media, Pd induces pronounced destabilization in its alloys, rendering it a surprisingly detrimental component of the HEA. In contrast, the enhanced stability of non-noble metals under alkaline conditions suppresses dissolution in the PdCuPtNiCo HEA even at a significantly higher potential, resulting in exceptional stability across a wide potential window. These findings underscore the importance of knowledge-driven materials design when combining noble and non-noble elements in HEAs. Overall, HEA formation enhances electrochemical stability compared to corresponding bimetallic systems: Pt is stabilized when alloyed with Ni or Co, whereas Pd introduces instability within multicomponent compositions.

## Graphical abstract

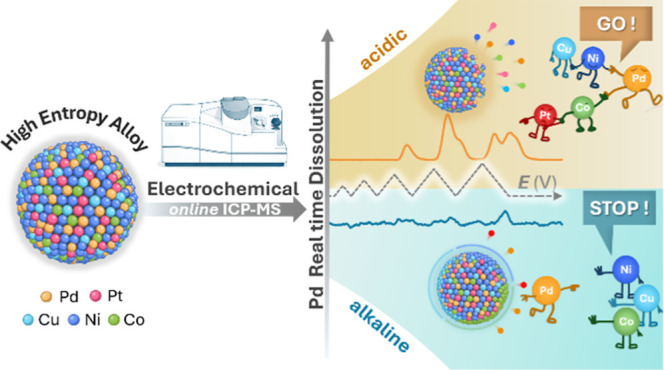

## Introduction

Rising global energy demands, fossil fuel depletion, and environmental concerns have intensified the need for renewable energy sources, such as wind and solar. However, their intermittency creates a supply-demand gap. To address this limitation, essential technologies include water electrolysis for hydrogen fuel production, rechargeable metal-air batteries, and reversible fuel cells. All of these technologies could contribute to a sustainable energy future. The advancement of energy conversion and storage systems is hindered by the slow kinetics of key electrochemical reactions, including the oxygen evolution reaction (OER),^1^ the oxygen reduction reaction (ORR),^2^ and the hydrogen evolution reaction (HER).^3,4^ Although platinum (Pt)- and noble-metal-based materials are currently the most practical electrocatalysts, their high prices and scarcity limit large-scale applications. Therefore, developing efficient and durable low-Pt-group-metal electrocatalysts for such processes is essential.

Combining Pt with non-noble metals has become a promising approach to both reducing catalyst costs and improving their performance.^5–7^ Taking the ORR as an example, recent research has focused on PtM catalysts, where M is typically Ni,^8,9^ Cu,^10,11^ Co,^12,13^ or Fe,^14^ and these PtM alloys often demonstrate comparable or enhanced activity to pure Pt. This performance is due to geometric and ligand effects resulting from composition-dependent interactions between the catalyst surface and adsorbates.^15^ Geometric effects involve changes in the arrangement of active sites, while ligand effects involve changes in surface electronics due to charge transfer between atomic centers. A representative example is a single crystalline Pt_3_Ni­(111) surface that exhibits exceptional ORR activity, performing 10 times better than a Pt(111) surface and 90 times better than a commercial Pt/C catalyst, which is attributed to the downshift of the d-band center and the weakened binding energies of the oxygenated species.^16,17^ Such improvements can be transferred to practical catalysts, such as 20 nm-sized alloy Pt_82_Co_18_ nanoparticles (NPs), which exhibit superior ORR performance compared to commercial Pt/C.^18^ This enhanced activity is due to the optimized binding strength of oxygenated reaction intermediates, i.e., *HO, *O, and *HOO. At the same time, the NPs were found to be structurally stable in terms of morphology, elemental distribution, and crystallinity, which was attributed to negligible electrochemical dissolution of the cobalt protected by a platinum shell during electrocatalysis.

The benefits offered by traditional alloy electrocatalysts have motivated the synthesis of high entropy alloy (HEA) NPs, which are defined as alloys composed of five or more principal elements in concentrations between 5 and 35 atomic percentage (at.%), resulting in high configurational entropy that stabilizes a single-phase solid solution.^19–21^ From a thermodynamic standpoint, HEA NP electrocatalysts could show remarkably high stability compared to monometallic and bimetallic alloy NPs due to their high configurational entropy (above 1.5R), preventing degradation processes such as the Kirkendall effect or dealloying.^22,23^ However, this assumption is rarely supported by data. For example, PdCuPtNiCo and PdCuPtNiFe NPs with high activity for the ORR were noted to have a 180% increase in activity after a 10 k cycle durability test.^24^ An increase in activity with use is typically indicative of structural or compositional changes, but the underlying reasons for the increased catalytic activity in this case remain unclear, although analysis by transmission electron microscopy (TEM) and elemental mapping indicated minimal loss of metal.

Electrocatalyst stability is influenced by parameters such as electrolyte composition, pH, temperature, pressure, and current density or potential as well as the composition of the catalyst itself. While the thermodynamic stability of metals is relatively easy to estimate, predicting kinetic contributions is challenging because of limited experimental data. For example, Pourbaix diagrams describe the thermodynamic stability of different phases of an element in aqueous media, identifying stable species at specific pHs and potentials.^25^ However, those diagrams do not provide kinetic information, a significant limitation as electrocatalytic reactions occur far from equilibrium and ideally at high current densities. Moreover, predicting the stability of the elements in the alloys becomes increasingly challenging as the number of metals involved increases.^26^ In Pt-based alloys, stability is strongly influenced by the dissolution of less noble metals such as Ni and Co under acidic conditions, restricting their content to optimized, limited levels in practical proton-exchange-membrane-fuel-cell (PEMFC) catalysts, such as those employed in the Toyota Mirai or Hyundai Nexo. Indeed, while alloying enhances ORR activity, the leaching of base metals and resulting morphological changes remain key degradation pathways.^27,28^ Interestingly, Pt-based alloys may offer greater promise under alkaline conditions, where such dissolution is less severe, although their long-term stability in base remains less well-understood. As discussed in our recent review, the mode of alloying plays an important role, which makes predicting the stability based on only the composition of the alloy unreliable.^26^

Consequently, benchmarking electrocatalyst stability becomes essential and typically involves accelerated stress or durability tests, which include potential cycling or chronoamperometric measurements and measuring activity with a linear sweep or cyclic voltammetry before and after many cycles (e.g., 20 k cycles). Ex situ characterization by scanning electron microscopy (SEM), TEM, energy-dispersive X-ray spectroscopy (EDX), X-ray photoelectron spectroscopy (XPS), and Raman spectroscopy often accompanies data from durability tests, but this characterization requires catalyst removal and sample preparation, potentially altering the surface composition. These methods also provide only indirect information about degradation mechanisms and on kinetic analysis.

Here, a scanning flow cell (SFC) is combined with an inductively coupled plasma mass spectrometer (online ICP–MS) to enable real-time quantification of dissolved metal ions released from NP electrocatalysts during electrochemical protocols.^29^ This technique allows simultaneous electrochemical measurements and detection of trace metal dissolution (down to picogram cm^–2^). To date, only studies on the dissolution of bimetallic and noble metal-only HEA systems have been reported.^30^ A systematic stability study of similarly sized and shaped bimetallic and HEAs containing both noble and non-noble metals is still missing. Specifically, the stability and dissolution behaviors of representative HEA PdCuPtNiCo NPs consisting of noble and non-noble metal elements were investigated and compared to PtPd, PtNi, and PtCo NPs. The different NP samples were synthesized with comparable size and monodispersity. Then, stability screening was performed for all the materials in a wide potential window using the SFC-ICP-MS setup. In order to assess the effect of alloying on the material’s stability under the most aggressive conditions for all the elements , the measurements were performed in both alkaline and acidic environments. The results show that alloying significantly impacts the electrochemical stability of transition metals. In acid, Pt shows enhanced stability when combined with Co or Ni, likely due to the formation of a protective Pt-rich shell. In contrast, Pd appears to destabilize the HEA, remaining at the surface and promoting the dissolution of acid-sensitive elements like Ni and Co. However, in alkaline media, the presence of stable elements such as Co, Ni, and Cu mitigates Pd dissolution, making HEA/C substantially more stable than PtPd/C. Our findings demonstrate that the incorporation of multiple elements into HEAs significantly alters their electrochemical stability and catalytic activity, revealing a rich and complex behavior that opens exciting avenues for further exploration.

## Experimental Section

### Materials

Palladium­(II) bromide (PdBr_2_), platinum­(II) bromide (PtBr_2_), copper­(II) acetate (Cu­(ac)_2_), nickel­(II) acetylacetonate (Ni­(acac)_2_), cobalt­(II) chloride hexahydrate (CoCl_2_·6H_2_O), platinum­(II) acetylacetonate (Pt­(acac)_2_), palladium­(II) acetylacetonate (Pd­(acac)_2_), cobalt­(II) acetate tetrahydrate (Co­(ac)_2_·4H_2_O), nickel­(II) acetate tetrahydrate (Ni­(ac)_2_ 4H_2_O), palladium­(II) acetate (Pd­(ac)_2_), oleylamine (OAm 70% O7805), oleic acid (90%, OA), trioctylphosphine (97%, TOP), 1,2-dodecanediol (90%, DDD), and 1-octadecene (90%, ODE) were purchased from Sigma-Aldrich. Hexane, ethanol, acetone, and toluene were of analytical grade. All chemicals were used without further purification. All syntheses were carried out using a vacuum/dry argon (Ar) Schlenk line. All glassware was washed with aqua regia and then distilled water several times.

The chemicals used in this work present a range of health, safety, and environmental hazards typical of transition-metal salts, organic ligands, and volatile solvents. Many of the metal precursors, particularly nickel and cobalt compounds such as nickel­(II) acetylacetonate, nickel­(II) acetate, cobalt­(II) chloride hexahydrate, and cobalt­(II) acetate, are classified as carcinogenic and strong skin or respiratory sensitizers, posing risks upon inhalation, ingestion, or skin contact. Organic reagents such as oleylamine are corrosive and can cause severe skin and eye damage, whereas trioctylphosphine is flammable, air-sensitive, and toxic. Solvents, including hexane, acetone, ethanol, and toluene, are highly flammable and may cause central nervous system depression, with hexane and toluene additionally being associated with neurotoxicity upon prolonged exposure. Thus, appropriate personal protective attire should be worn, and nanoparticle syntheses should be conducted in a fume hood.

### Synthesis of PtNi NPs

Bimetallic NPs were prepared by slow injection of precursor solution into a heated solvent.^31^ Specifically, Ni and Pt precursor solutions were prepared by dissolving 12.93 mg and 20.73 mg of the respective salts (Ni­(ac)_2_·4H_2_O for Ni and Pt­(acac)_2_ for Pt) in 5 mL of OAm (70%) each. The solutions in the scintillating vials were stirred for 1 h at 100 °C on a heat plate until the salts were completely dissolved in OAm. Subsequently, 1 mL of each precursor solution was combined in a septum-capped vial and stirred for 30 min under a vacuum. Meanwhile, in a 50 mL three-neck round-bottom flask (RBF) equipped with a reflux condenser, thermocouple adapter, rubber septum, and magnetic stir bar (octagon), 10 mL of ODE and 6 mL of OAm were mixed. The flask was evacuated, heated to 120 °C, and maintained for 30 min before undergoing Ar purging. Under an Ar atmosphere, the flask was then heated to 275 °C. The precursor solution mixture of 2 mL (1 mL of Pt and 1 mL of Ni) was injected slowly at 0.4 mL min^–1^ using a syringe pump into the solvent flask at the reaction temperature. After addition, the reaction proceeded for 5 min. Upon completion of the reaction time, the flask was allowed to cool. The product was isolated by washing the particles three times with a 1:4 ratio of toluene and acetone, followed by centrifugation at 11,404*g*. The final product was suspended in toluene.

### Synthesis of PtCo NPs

PtCo NPs were synthesized similarly to the previously mentioned PtNi NPs. Here, the Co precursor solution was prepared by dissolving 12.37 mg of CoCl_2_·6H_2_O in 5 mL of OAm. After the addition of the Pt and Co precursor solution to the heated solvent, the reaction proceeded for 15 min, and the product was washed in a similar way.

### Synthesis of PtPd NPs

PtPd NPs were synthesized using a method similar to that for PtNi and PtCo NPs. Here, the Pd precursor solution was made using 16 mg of Pd­(acac)_2_ in 5 mL of OAm, and the reaction proceeded for 10 min. The product was washed and stored in a similar fashion.

### Deposition of NPs on Ketjen Carbon

The alloy NPs were deposited on carbon support (Ketjen EC-600JD) by considering a mass ratio of 1:10. The NPs were measured using a weighing balance after drying the solvent. After weighing, the dried NPs were redispersed in hexane. Meanwhile, the required amount of carbon was added to a 10 mL scintillating vial and sonicated with 5 mL acetone and 2.5 mL hexane. The NP solution was then added dropwise to the carbon slurry, stirring at 800–1000 rpm with a stir bar. The addition rate 10 μL per 5 s and a syringe pump was used to add the NPs solution to the carbon slurry. Once the NPs were added, the solution was sonicated for 1 h and then left to stir overnight at 800–1000 rpm.

### Synthesis of PdCuPtNiCo NPs

HEA PdCuPtNiCo NPs were prepared by a three-step process: NP seed synthesis, core–shell NP synthesis, and thermal annealing.^24,32^ First, PdCu NPs were synthesized using a previous method for use as seeds.^33^ To a 100 mL RBF, 36 mg of Cu­(ac)_2_, 54 mg of PdBr_2_, 18 mL of OAm, and 40 μL of OA were added, and this mixture was heated under vacuum to 110 °C with a heating mantle and held for 10 min with stirring. Then, 60 μL of TOP was added, and the solution was heated to 235 °C under Ar and held at this temperature for 30 min. Then, the solution was cooled to 70–80 °C, and 40 mL of hexane was added. Then, the total solution was separated into two centrifuge tubes and centrifuged at 2050*g* for 10 min to remove any large particles. The rest of the particles (contained in the collected supernatant) were precipitated by adding a mixture of 3:1 acetone/ethanol, followed by collection by centrifugation at 11,404*g* for 10 min. The obtained particles were centrifuged again at 11,404*g* for 10 min with a mixture of ethanol and hexane (5:1 vol). After removal of the supernatant, the particles were finally resuspended in 3 mL of hexane and 16 mL of a solution of acetone/methanol 2:1 for storage.

To synthesize core–shell PdCu@PtNiCo NPs, bimetallic PdCu cores (0.02 mmol–0.06 mmol) were added to a 100 mL three-neck RBF containing 4 mL of OAm (70%), 5 mL ODE, PtBr_2_, Ni­(acac)_2_, and Co­(ac)_2_·4H_2_O. The concentrations of the metal precursors were calculated by considering a molar ratio of 1:1 of [seed/core] to [metal precursors]. 1,2-Dodecanediol was added with a 20:1 DDD: [seed/core] at the same time. This reaction solution was first stirred at room temperature for 10 min and then heated to 110 °C under a vacuum and held at that temperature for 30 min. Then, the temperature was increased to 235 °C under an Ar blanket and was allowed to incubate for 30 min. The solution was allowed to cool down to 70–80 °C and centrifuged at 2050*g* for 10 min to remove any large particles. The rest of the particles (contained in the collected supernatant) were precipitated by adding a mixture of 3:1 acetone/ethanol, followed by collection by centrifugation at 11,404*g*. After the removal of the supernatant, the particles were finally resuspended in hexane for further use.

In the third step of annealing, first, the core–shell NPs were dispersed on a carbon support (Ketjen) (1:10 NP: carbon mass ratio) with 5 mL acetone and 2.5 mL hexane. The solvents were then evaporated with N_2_ flow, and the resulting solids were dissolved in 1–2 mL of acetone by sonication and transferred onto a quartz crucible. The crucible was centered in a fused silica tube and heated in a tube furnace. The fused silica tube was purged with an H_2_/N_2_ (4 v./v.%) mixture for 30 min, and then the samples were heated to 600 °C for 10 h at a ramp rate of 35 °C min^–1^. Finally, the carbon-supported HEA NPs were stored in a dry glass vial.

### Characterization

Transmission electron microscopy (TEM) images of NPs were obtained on a JEOL 1400Plus operating at 120 keV, and images were collected with a CMOS camera (Oneview camera, Gatan). Scanning TEM energy dispersive X-ray spectroscopy (STEM-EDS) images were collected with JEOL JEM 3200FS microscope operating at 300 keV using a 4 k × 4 k GatanUltraScan 4000 CCD camera. The JEOL JEM 3200FS was interfaced with an Oxford Silicon Drift Detector for EDS. All samples for TEM and STEM imaging were prepared by drop casting ∼10 μL of solution onto carbon-coated Cu or Au grids (Formvar/Carbon 200 mesh Ted Pella). The grids were selected to ensure that the grid metal, and the metallic constituent of the analyzed NPs do not match.

The atomic percentages were determined by scanning electron microscopy (SEM-EDS) over a sample area of at least 10 μm^2^ (a large number of NPs) with the Zeiss Auriga 60 FIB-SEM equipped with the X-Max 50 mm^2^ silicon drift detector and the AZtec software package (Oxford Instrument).

The X-ray powder diffraction (XRD) patterns were collected on a PANalytical Empyrean instrument (operated at 40 kV and 45 mA) with Cu Kα radiation with a wavelength of 1.5418 Å and an X’Celerator linear strip detector. Samples for XRD were prepared by drop-casting NPs solutions onto single-crystalline Si substrates and allowing them to dry before analysis.

X-ray photoelectron spectroscopy (XPS) measurements were collected using a PHI 5000 Versa Probe II equipped with a focused monochromatic Al Kα X-ray source. A beam size of about 100 μm and an X-ray power of 50 W at 15 keV under ultrahigh vacuum conditions were used for all experiments. Binding energies were calibrated by assigning the C 1s peak to 284.8 eV.

The sizes of the NPs were measured using ImageJ or FIJI. More than 100 NPs were measured per sample to calculate the standard deviations of size.

### Stability Measurements (Online ICP–MS)

The suspensions of the synthesized NPs were prepared with ultrapure water (Milli-Q IQ 7000 Merck) and 2-propanol (Emsure, Merck, ≥99.8% purity) in a ratio of 7:1. Nafion (Sigma-Aldrich, 5 wt %) was added to the suspension as a binder in the amounts for the total Nafion content to be 20% of the ink. The suspension was sonicated with the sonication horn (Branson SFX 150) for around 20 min with intervals (4 s pulse, 2 s pause) and 40% intensity until the ink was homogeneous. To prevent the heating of the ink mixture, the vial was kept in an ice bath during sonication. After sonication, the pH of the suspension was adjusted to ∼10 with 1 M KOH before drop-casting 0.25 μL of the suspension on a freshly polished glassy carbon (GC) plate (5 × 5 cm^2^, Sigradur G, HTW), serving as a working electrode. The loading of the catalysts was aimed to be 20–25 μg cm^–2^ in total. Considering the loading of the metal NPs on the carbon black, the total loading of the metals was 2–2.5 μg cm^–2^. The quality and the area of the drop-casted spots were examined with the use of a laser microscope (Keyence VK-X250). The working electrode was placed on a translational stage (Physik Instrumente M-403), allowing it to move along the electrode and quickly screen multiple samples. All electrochemical measurements were performed using a Gamry Reference 600 potentiostat. All instruments (gas control box, mass flow controllers, peristaltic pump, and translational stage) were controlled by homemade LabView software.

The stability of the drop-casted samples was examined using a scanning flow cell (SFC) combined with inductively coupled plasma mass spectrometry (ICP–MS).^29^ A GC rod and an Ag/AgCl electrode (Metrohm) were used as counter and reference electrodes (CE and RE), respectively. Note that all potentials are reported on the reversible hydrogen electrode (RHE) scale. Freshly prepared 0.05 M H_2_SO_4_ (96% Suprapur, Merck) or 0.1 M KOH (Suprapur, Merck), saturated with Ar, was used as an electrolyte and pumped through the setup with a flow rate of 3.5 ± 0.2 μL s^–1^. The electrolyte flow rate was controlled by the peristaltic pump of the ICP–MS (Elemental Scientific M2) and calibrated daily. The ICP–MS (PerkinElmer NexION 350× ICP–MS) instrument was calibrated daily with known amounts of analyzed metals (Co, Ni, Cu, Pt, and Pd) and internal standards (^187^Re and ^74^Ge). Both electrochemical and ICP–MS results were normalized by the geometric surface area of the drop-casted catalyst spots.

Electrochemical protocols A and B were performed in this study to assess the stability of the catalysts:

*Protocol A* included the following steps: (i) initial contact at 0.05 *V*_RHE_ and hold for 5 min, (ii) a series of CVs from 0.05 *V*_RHE_ to 0.6, 0.9, 1.2, 1,5, and 1.8 *V*_RHE_ at the scanning rate of 10 mV s^–1^, and finally (iii) holding at 0.05 *V*_RHE_ for 3 min;

*Protocol B* was based on the potentiostatic holds starting with (i) holding at 0.05 *V*_RHE_ for 5 min, followed by (ii) holding at 1.2 *V*_RHE_ for 5 min, and (iii) holding at 0.05 *V*_RHE_ for 5 min.

Note that no additional steps other than those described in the synthesis sections were undertaken to remove residual capping ligands; however, the initial contact holds are anticipated to remove any remaining capping ligands.

## Results and Discussion

### Phase Purity and Elemental Distribution in Pt-Based Alloys

Four different alloy NPs (PtPd, PtCo, PtNi, and PdCuPtNiCo) loaded on carbon were synthesized by colloidal methods, and their durability was evaluated compared to commercial Pt NPs. Figure 1 shows the morphological and elemental characterization of these NPs, along with the commercial Pt NPs in Figure S1. PtPd, PtNi, and PtCo NPs were obtained by direct coreduction of metal precursors slowly injected (0.4 mL min^–1^) into a heated reaction medium composed of OAm and ODE. TEM images reveal that the average diameters of the PtPd, PtCo, and PtNi NPs are 7.3 ± 1.4 nm, 12.7 ± 1.5 nm, and 13.0 ± 3 nm, respectively (Figures 1a, e, i, S2a, g, m), indicating relatively monodisperse distributions for the three bimetallic systems but with some slight variations in NP size. STEM-EDS mapping and line-scan analyses show homogeneous elemental distributions at the nanoscale across (Figures 1b,c, f,g, j,k, S2b–e, h–j, n–p), with no evidence of core–shell NP architectures or segregated phases.

**Figure 1:**
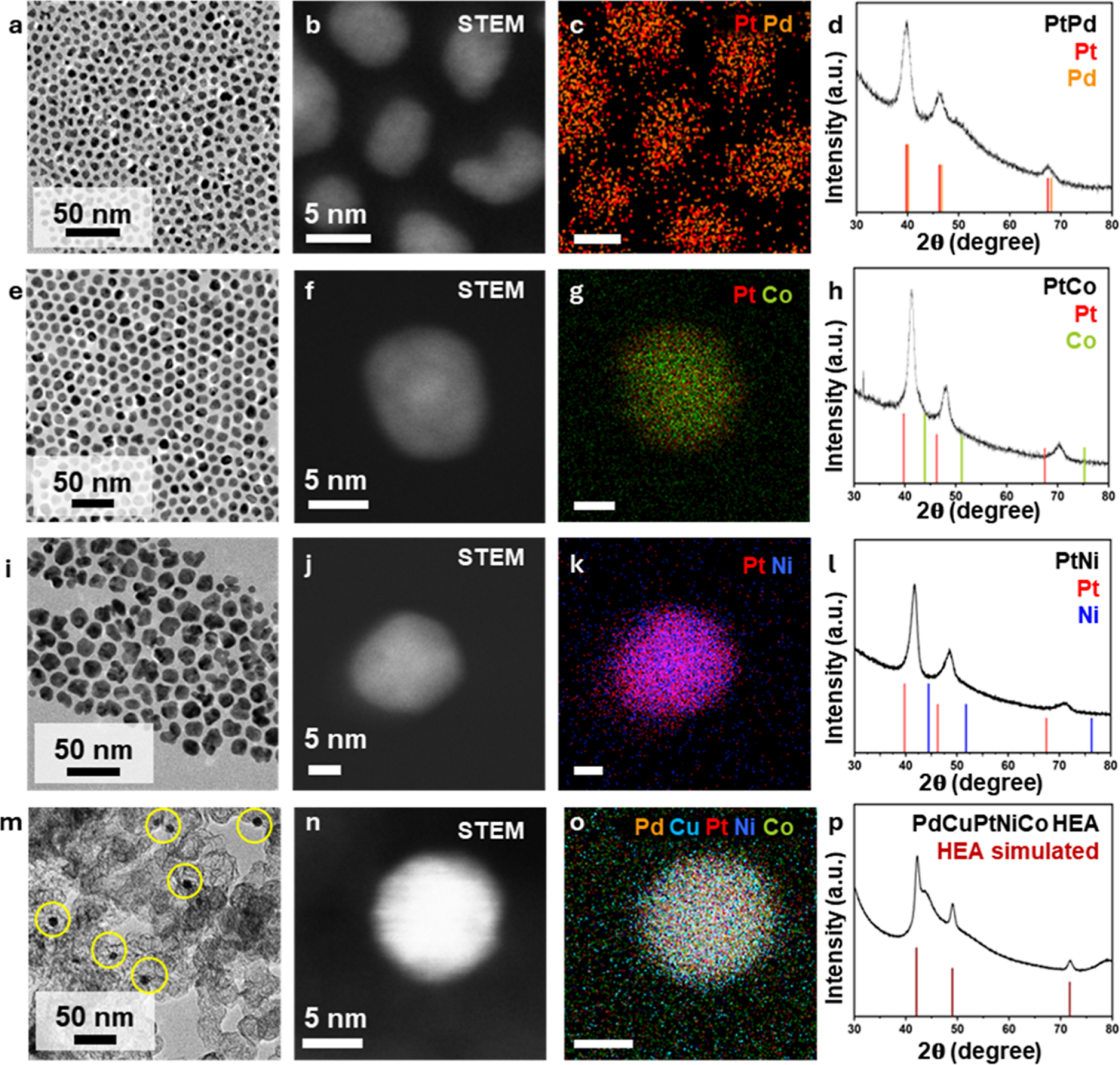
TEM images, STEM and EDS elemental mapping images, and XRD patterns of the samples (a–d) PtPd, (e–h) PtCo, (i–l) PtNi, and (m–p) PdCuPtNiCo/C NPs. The yellow circles in (m) denote the PdCuPtNiCo NPs. References: Pt (ICSD: 243678), Ni (ICSD: 260172), and Co (ICSD: 52934). XRD HEA alloy references were calculated by considering the elemental composition by SEM-EDS and Vegard’s law.

Bulk elemental analysis using SEM-EDS gave the following compositions: PtPd equals 31:69, while PtCo and PtNi exhibit approximately 49:51 and 53:47 Pt-to-base-metal ratios, respectively (Figure S2f, l, r). Powder XRD patterns for the bimetallic systems indicate single-phase face-centered cubic (fcc) structures with peak positions intermediate between those of the pure constituent metals, suggesting successful alloy formation without phase separation (Figure 1d, h, and l).

PdCuPtNiCo HEA NPs were synthesized using a previously reported NP conversion method, where colloidally prepared core–shell NPs were dispersed on a carbon support and transformed into HEA NPs through thermal annealing (Figures S3,S4).^24,34–37^ A TEM image shows that the HEA NPs have uniform sizes around 11.8 ± 2.5 nm (Figure 1m). STEM-EDS elemental mapping indicates homogeneous mixing of all five metals (Figures 1n,o, S5). The XRD pattern exhibits a single set of fcc peaks, indicating the formation of a single-phase solid solution (Figures 1p, S6). The predicted XRD pattern using Vegard’s law and SEM-EDS is in agreement with the PdCuPtNiCo XRD pattern. The bimetallic and HEA NPs are indicated by “/C” in their names in the following sections when dispersed onto a carbon support.

### Dissolution of Pt-Based Alloys in Acidic Electrolyte

The stability of Pt-group metals at low pH was studied extensively in previous reports.^38–41^ Here, we examine the electrochemical stability of PtPd/C, PtCo/C, PtNi/C, and PdCuPtNiCo/C electrocatalysts in acidic media to assess the impact of non-Pt-group metals and entropic stabilization on electrocatalyst stability. Moreover, we compare the dissolution trends to commercial Pt/C. A wide potential window (from 0.05 *V*_RHE_ as the low potential limit (LPL) to 1.8 *V*_RHE_ as the maximal upper potential limit (UPL)) was chosen for the electrochemical protocols. Such a wide potential window allows us to assess the stability of the materials under various conditions for diverse applications. Specifically, to analyze the stability of the catalysts and track their dissolution, we used the online ICP–MS technique.^29^ First, we developed a protocol to follow the materials’ dissolution during a dynamically changing potential by performing cyclic voltammetry (CV), where each following cycle had a higher UPL, which varied from 0.6 *V*_RHE_ to 1.8 *V*_RHE_ (*Protocol A*, Figure 2). Varying the upper potential in the CV experiments allowed us to identify the potentials at which platinum oxide species form and significant dissolution happens. The CVs were performed at a relatively low scan rate of 10 mV s^–1^ in order to separate possible anodic and cathodic dissolution peaks.

**Figure 2:**
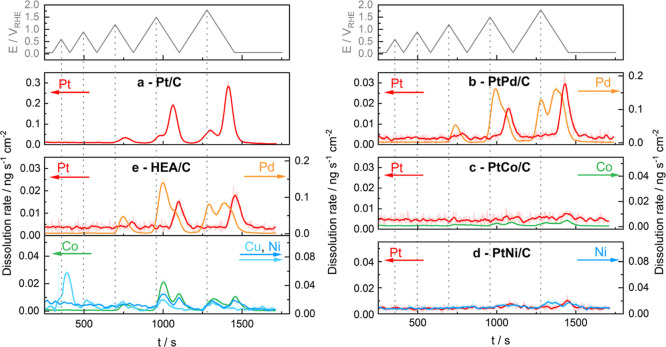
Stabilization effect of the alloying on the durability of metals in 0.05 M H_2_SO_4_. Applied protocol (*Protocol A*) and the dissolution profiles of (a) Pt/C, (b) PtPd/C, (c) PtCo/C, (d) PtNi/C, and (e) PdCuPtNiCo/C (HEA/C). The first 250 s (contact dissolution peaks, Figure S13) were excluded from this figure for clarity.

Figure 2 depicts the dissolution profiles of Pt/C (a), PtPd/C (b), PtCo/C (c), PtNi/C (d), and HEA/C (e) in 0.05 M H_2_SO_4_ (pH ∼1) recorded for *Protocol A*. Although Pt dissolution at this pH is not predicted by the thermodynamic data presented in the Pourbaix diagram (Figure S7), where no soluble species are presented at these potentials and this pH, the observed dissolution can be explained by the transient processes, as reported in the previous studies.^38^ The dissolution profile of Pt/C in acidic media (Figure 2a) is similar to those reported in the literature.^38–40,42^ According to the Pourbaix diagram (Figure S7), the potential of Pt­(OH)_2_ and Pt oxide species formation at pH 1 is ca. 0.96 *V*_RHE_ and 1.06 *V*_RHE_, respectively.^42^ The observation of no Pt dissolution for the first two cycles is consistent with the Pourbaix diagram, as the potential does not reach the critical values. These oxidation processes do not cause noticeable anodic dissolution, also during the third cycle, where the UPL is 1.2 *V*_RHE_. This UPL is, however, high enough to form a sufficient amount of the oxidized species whose reduction during cathodic sweep results in the observed dissolution peak. A recent study revealed a correlation between Pt-oxidation/reduction at varied potentials and Pt dissolution.^43^ Depending on the potential, different processes occur during the anodic sweep, which affect Pt dissolution in different ways. Specifically, at lower potential, the anodic Pt dissolution has been associated with a surface oxide formation and the place-exchange-like mechanism between surface Pt atoms and oxygenated species, leading to transient surface reconstruction and Pt detachment.^42,44^ While higher potentials, ordered oxide layers can passivate the surface, or metastable intermediate oxide structures can momentarily enhance the solubility under nonequilibrium conditions, leading to dissolution.^42^ The overall correlation between the UPL and the intensity of Pt dissolution, however, is, straightforward. The increase in the anodic and cathodic dissolution is further observed when the potential exceeds 1.2 *V*_RHE_. Moreover, the dominance of the cathodic dissolution confirms that the dissolution occurs due to transient processes such as the reduction of previously formed oxidized platinum species. This finding agrees with previously published works.^41,42,45,46^ When the anodic potential is high enough to form oxides, the dissolution also depends on the lower potential limit – as this potential increases, the formation of a passivating oxide layer suppresses dissolution. Previously, the onset of platinum oxide reduction in acidic electrolyte was estimated to be around 1.05 *V*_RHE_.^38,41^ Here, we set the LPL (0.05 *V*_RHE_) low enough and kept it constant in contrast to the varied UPLs to ensure that a full reduction of the formed platinum oxide species occurs.

The dissolution rate of Pt in PtPd/C is approximately ten times lower compared to the Pt/C commercial sample. However, due to the differences in morphology, particle size, and surface area, it is inappropriate to compare the dissolution of the synthesized NPs and the commercial Pt/C quantitatively. Therefore, here, we will refer only to the observed changes in the dissolution trends. Interestingly, alloying Pt with Pd leads to almost complete disappearance of the anodic Pt dissolution (Figure 2b). Cathodic dissolution does not seem to be affected, which means that the presence of Pd does not prevent Pt-oxidation but rather suppresses the transient anodic dissolution caused by this process. Notably, the dissolution processes of Pd and Pt are independent from each other, as the start of dissolution differs drastically for both anodic and cathodic processes (Figures 2b and 3). The Pd dissolution peaks display higher intensities in both the anodic and cathodic sweeps, indicating an intense dissolution compared to Pt (Figure 2b), which aligns well with previous studies of pure Pd.^47^ According to the Pourbaix diagram presented in Figure S8, two oxidation processes occur in the explored potential window–formation of Pd^2+^ species (dissolved ions at pH 1), followed by the formation of the Pd^4+^ species (in the form of stable Pd­(OH)_4_). Similar to Pt, these oxidation reactions result in noticeable anodic dissolution only at much higher potentials, while cathodic dissolution is pronounced starting with the UPL of 1.2 *V*_RHE_. This suggests that Pt stabilizes Pd, moving the onset of anodic dissolution to higher potentials. Reduction of the previously oxidized Pd species results in two cathodic dissolution peaks that overlap starting with the UPL of 1.5 *V*_RHE_. Notably, the anodic and cathodic peaks are clearly separated only in the last cycle with the UPL of 1.8 *V*_RHE_.

**Figure 3:**
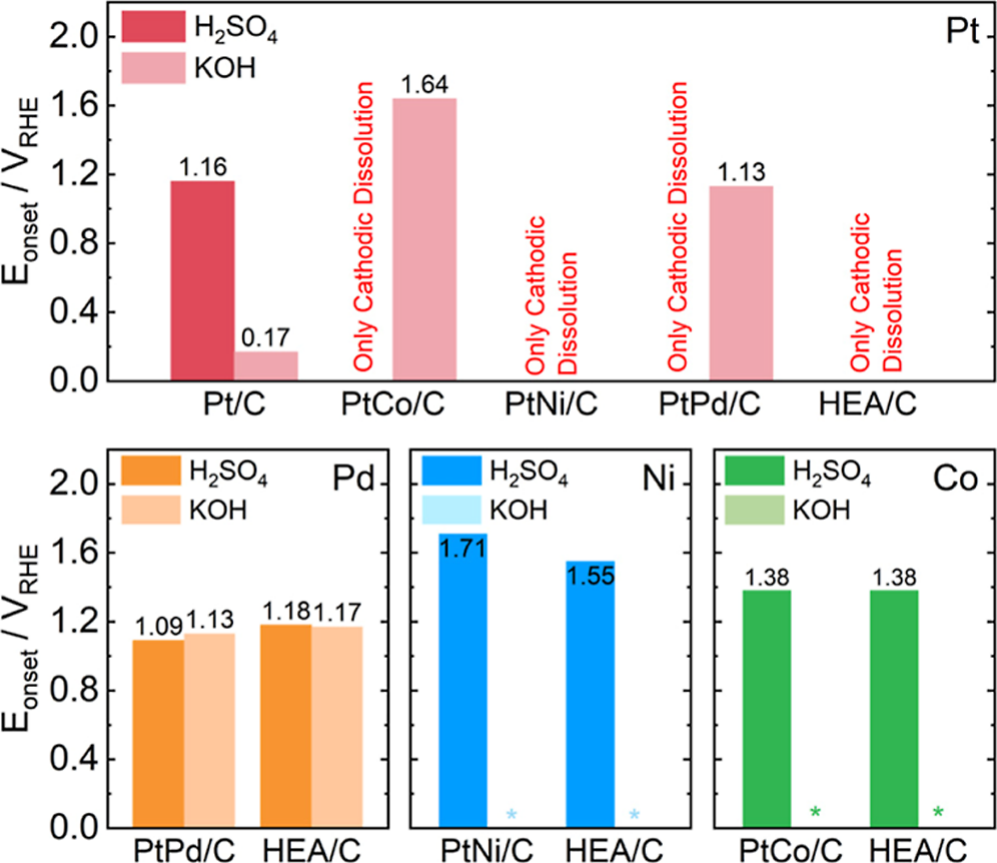
Onset potential of dissolution in the samples in acidic and alkaline electrolytes. Here, we present the onset potential of anodic dissolution, as it is challenging to obtain the cathodic dissolution onset due to the overlap of the dissolution peaks. In the cases of only cathodic dissolution, it is stated in the figure. The asterisk sign means that no dissolution was observed, or the dissolution of these elements was below the detection limit of ICP–MS.

Surprisingly, the dissolution of Pt in acidic media decreases significantly for PtNi/C and PtCo/C (Figure 2c,d), suggesting that the stabilization effect plays a role not only for the unstable non-noble metals in acidic media but also for Pt. No significant anodic or cathodic dissolution was registered for Pt in these bimetallic alloys, which might be explained by either suppression of its redox reactions or surface passivation. On the other hand, Ni and Co also exhibit lower dissolution than was previously published and expected from the thermodynamics data presented in Figures S9–S11.^48,49^ Co dissolution depends on the UPL, as there are both anodic and cathodic dissolution peaks of low intensity when the UPL is higher than 1.2 *V*_RHE_ (Figure 2c). Ni dissolution, however, is minimal even during the CVs with high UPLs (Figure 2d). One explanation for the extremely low dissolution of PtNi/C could be its flat CV shape (Figure S12). Compared to the other alloys, PtNi/C has almost indistinct redox features and, therefore, insignificant charge accumulation or transfer from the electrochemical redox reactions, which do not give rise to drastic transient dissolution. In the case of PtCo/C, we observe much more pronounced redox peaks for the CVs with the UPL higher than 1.2 *V*_RHE_ (Figure S12), which is consistent with the dissolution profiles (Figure 2c). Another explanation for the lack of significant dissolution of non-noble metals in an acidic solution could be that these elements were removed from the surface of the catalyst during ink preparation and/or the initial electrode/electrolyte contact, which usually causes drastic dissolution.

To examine this possibility, we plotted all the contact peaks collected during all the measurements in acidic electrolyte for both protocols (Figure S13). Usually, the contact peak is not analyzed because it is challenging to predict such dissolution or draw conclusions based on this data, as electrode aging during air exposure and dissolution during ink preparation can affect the dissolution during the first contact with the electrolyte.^50^ In our case, however, we integrated the contact peaks to evaluate how much of each metal was removed from the catalyst’s surface before the start of the main protocols. By examining the amount of dissolved metals (ng cm^–2^, Figure S14), we can predict the formation of the core–shell particles during the electrode/electrolyte contact for PtNi/C. Indeed, Pt dissolution is almost six times lower than Ni dissolution, suggesting the formation of a Pt-rich shell around the PtNi core during the hold before the start of the cycling. The formation of a Pt-rich shell explains such improved stability of both Pt and Ni and correlates well with previously published works on PtNi alloys, even though such low dissolution has not yet been reported.^51,52^ Interestingly, the contact dissolution in the case of PtCo/C does not allow us to draw a similar conclusion, as the dissolution rates of both metals are similar.

To judge whether there are any non-noble metal atoms left on the surface, we estimated the number of atoms present on the surface of the NPs in the measured spots and compared it with the estimated number of atoms removed from the measured spot due to the contact dissolution (Table S1). As one can see, these numbers support our hypothesis about the Pt-rich shell formation; almost all of the Ni atoms from the PtNi/C surface were removed during the contact peak. A 2 orders of magnitude higher number of Pt atoms were left on the surface. In the case of PtCo/C, the situation is similar, but the dissolution of Co from the surface of PtCo/C NPs was not so severe, and many more Co atoms are still estimated to be present in the Pt-rich shell (Table S1).

As the non-noble metals in the bimetallic alloys exhibited stability in acidic media and even led to the significant stabilization of Pt, identifying whether alloying them all together, not only with Pt but also with Pd, is important to advanced electrocatalyst design. For the HEA PdCuPtNiCo/C, the dissolution of Pt lowers slightly compared to that in the PtPd/C sample but does not disappear as was observed for PtCo/C and PtNi/C (Figure 2e). Pd dissolution in both PtPd/C and PdCuPtNiCo/C exhibits similar behavior (Figure 2b and e). Here, a large difference in anodic dissolution for the last two CVs is visible–both anodic and cathodic peaks are lower for the CV with the UPL of 1.8 *V*_RHE_ than for the CV with the UPL of 1.5 *V*_RHE_. Surprisingly, Pd seems to be the least stable element in this HEA in acidic electrolyte and not only does not seem to be stabilized by other elements but also affects the stability of Co and Ni, as their dissolution profiles in Figure 2e are similar. Co and Ni atoms seem to leach into the electrolyte dragged by dissolved Pd atoms. While Pt, Ni, and Co are all stabilized in their bimetallic alloys, as was mentioned above, Pd destabilizes the HEA’s structure. According to Figure S14, the dissolution rates of Pt and Pd in the PdCuPtNiCo/C during the contact hold are the lowest. Based on this observation, we can assume the formation of Pt–Pd-rich shells around the HEA cores. Mechanistically, Pd dissolves and drives the dissolution of Co and Ni not only from the surface but also from the cores, destabilizing the alloy. This is again supported by our estimation in Table S1, as the number of dissolved Ni atoms is higher than what could possibly be present on the surface. Therefore, it is safe to assume that Ni started leaching from the core of the NPs already during the initial contact between the NPs and the electrolyte. This is not observed for any of the bimetallic alloys, which only supports our conclusion of Pd’s destabilizing effect in HEA in acidic media. Additional evidence for that appears if we compare the shape of the anodic and cathodic peaks for Co and Ni. One can see that for the CVs with the UPL ≥1.5 *V*_RHE_, the first cathodic peak resembles Pd dissolution, while the second one replicates the shape and the position of the Pt cathodic peak. A further destabilizing factor may be the presence of Cu atoms in the shell, as Cu dissolution during the contact was estimated to be relatively low despite the predicted instability (Figure S11). This instability can contribute to the degradation of the HEA, as significant Cu dissolution is triggered at the beginning of the cycling (cathodically, after 0.6 *V*_RHE_ UPL), while all the other elements are still stable. The destabilization of this HEA is also confirmed by the estimated onset potentials of dissolution (Figure 3), as Ni in PdCuPtNiCo/C starts to dissolve at lower potentials during the anodic sweep.

As the UPL of 1.2 *V*_RHE_ appears to be the borderline potential, after which we observe the cathodic dissolution due to the reduction of the oxidized species, we developed a second protocol. In *Protocol B* (Figure S15), we exposed the samples to potentiostatic holds (5 min each) and switched the potential from reducing (0.05 *V*_RHE_) to oxidizing (1.2 *V*_RHE_) and back to separate the anodic and cathodic dissolution peaks. In contrast to the behavior during the dynamic potential change, a sudden change in the potential from 0.05 to 1.2 *V*_RHE_ causes significant anodic dissolution of Pt in Pt/C and Pd in PtPd/C, respectively (Figure S15a,b). Such intense anodic dissolution without significant cathodic dissolution for Pd contradicts what we observed for *Protocol A* but is consistent with published results for Pd thin films obtained under *Protocol B*.^30,53^ In this case, the reduction processes do not cause dissolution of Pd in PtPd/C. The behavior of Pt is similar to that observed for *Protocol A*, without clear anodic or cathodic dissolution peaks for Pt in PtPd/C, PtCo/C, and PtNi/C (Figure S15c,d). This behavior is consistent with the calculated onset potentials of dissolution present in Figure 3, which predict that Pt dissolution in these alloys (PtPd/C, PtCo/C, and PtNi/C) does not occur during anodic sweeps, where even the dissolution of non-noble metals occurs only at much higher potentials. Different behavior is observed for the anodic dissolution of Co and Ni in PdCuPtNiCo/C. During the dynamic protocol, Co and Ni exhibit quite high onset potentials for dissolution during the anodic sweep, higher than 1.3 *V*_RHE_, even in PdCuPtNiCo/C. However, when the potential change is sudden (*Protocol B*, Figure S15), 1.2 *V*_RHE_ is enough to trigger anodic dissolution of not only Pd but also Co, Ni, and Cu. Sudden anodic dissolution of Pd triggers the dissolution of Co and Ni, most likely due to the destruction of the PtPd-rich shell formed during the initial electrode/electrolyte contact and additional destruction of the HEA core, as discussed above. Moreover, the Co dissolution profile repeats the dissolution profile of Pd during the anodic and Pt during the cathodic reactions. The dissolution of Ni and Cu, although triggered by the sudden anodic change in potential, appears to be independent of the other elements (Figure S15e). Cathodic dissolution of Pd and Co results in the cathodic dissolution of Pt, which is a unique behavior compared to the other alloys here.

When discussing the dissolution, one should consider not only the trends and the onset potential of dissolution but also the total dissolution obtained during various electrochemical protocols. Moreover, the dissolution normalized by the fraction of a given element in the alloy is important. This analysis can identify whether metal dissolution is due to a low amount of a given element on the surface or due to a given element’s intrinsic stabilization.

Figure 4 shows the total and normalized (by the atomic percentage of each element) dissolution of Pt, Pd, Co, and Ni in the alloys. The catalysts dissolve more intensely during the potentiodynamic protocols (*Protocol A*, Figure 4a) rather than potentiostatic holds (*Protocol B*, Figure 4b). Pt, Ni, and Co dissolve much less in the bimetallic alloys, even when they are not normalized by their content in the material. This finding is consistent with our assumption about the destabilizing role of Pd in PdCuPtNiCo/C in an acidic electrolyte. As non-noble metals are highly stable at high pH, assessing the stability trends of these alloys in alkaline media would be the next step in this work.

**Figure 4:**
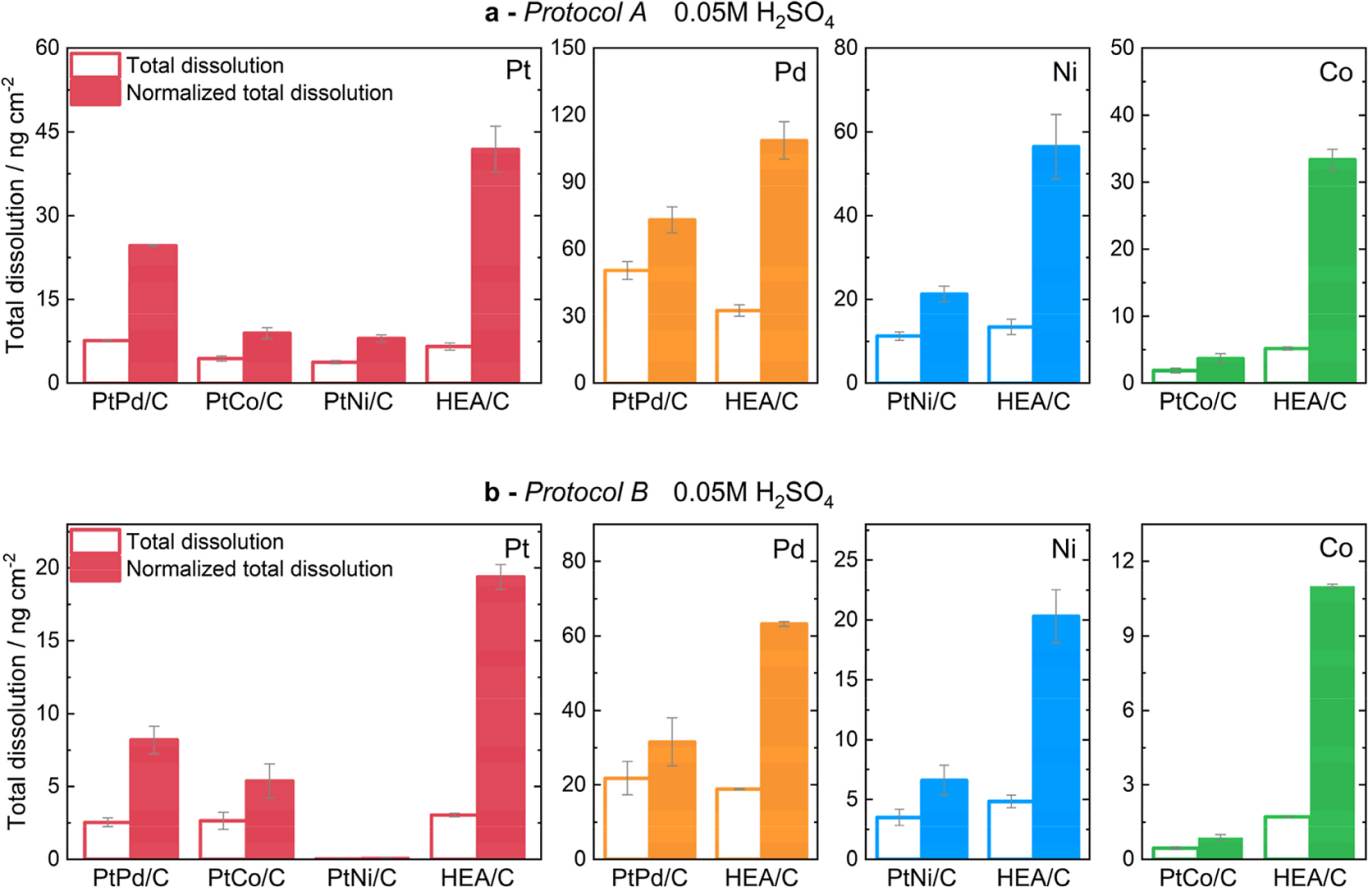
Total and normalized dissolution of the materials during (a) *Protocols A* and (b) *B* in acidic electrolyte (without the contact peak). Cu dissolution is not shown, as there is no reference point. The dissolution of Cu during *Protocol A* is 113.3 ± 21.10 ng cm^–2^; during *Protocol B*, it is 66.32 ± 13.64 ng cm^–2^.

Beyond pH, specific anion effects can also influence the dissolution behavior. Adsorbing anions, such as sulfate, can modify the stability of the surface sites. In particular, phosphate species have been reported to alter transient processes at varied pH values, including enhanced cathodic dissolution and suppressed redeposition during potential cycling.^42^ These effects are typically associated with differences in anion adsorption strength, surface coverage, and interactions with oxidized metal species. However, given the focus of the present study, such anion-specific contributions were not systematically addressed. Further dedicated experimental studies will be necessary to clarify these effects and their mechanisms.

### Dissolution of Pt-Based Alloys in an Alkaline Electrolyte

Figure 5 reports the dissolution profiles for the same alloys during *Protocol A* in 0.1 M KOH (pH 13). Overall, the dissolution trends of Pt in alkaline media for commercial Pt/C and the bimetallic alloys (PtPd/C, PtCo/C, and PtNi/C in Figure 4a) are similar to those in acidic electrolyte–Pt dissolution during the cathodic sweep is dominant. In the case of a 0.1 M KOH electrolyte, the predicted onset of oxide formation shifts to a much lower potential in terms of the SHE scale (Figure S7). However, the transition lines (formation of the platinum-oxidized species) follow the RHE lines for the HER and the OER (−59 mV/pH shift). Therefore, these oxidation processes and the formation of the Pt oxidized species are pH-independent and occur approximately at the same potentials at both low and high pH.^42^ The formation of various oxidized species that are not depicted in the Pourbaix diagram is proposed in the literature, e.g., Pt^4+^ in acidic media, as was already mentioned, and Pt­(OH)_4_^2–^, PtO_2_^2–^, or PtO_3_^2–^ in alkaline media.^42,44,46,54^ As discussed, previous works correlated the oxidation and reduction processes with the dissolution of Pt. We expect similar trends to take place in alkaline electrolyte.^38,43^ The anodically formed (hydr)­oxides play a crucial role in the stability of Pt, as their reduction during the cathodic sweep causes the transient dissolution of Pt. Therefore, cathodic dissolution dominates when the UPL is high enough to form these species. Indeed, Pt dissolution in Pt/C starts at quite low potentials (Figure 3). Similar to acid, Pt dissolution in base becomes more obvious when the UPL in the CVs increases, and more oxide species are formed on the catalyst’s surface. This observation agrees with the previously published works, where the authors showed that Pt cathodic (and overall) dissolution is higher when higher anodic potentials are applied.^41,42,45,46^

**Figure 5:**
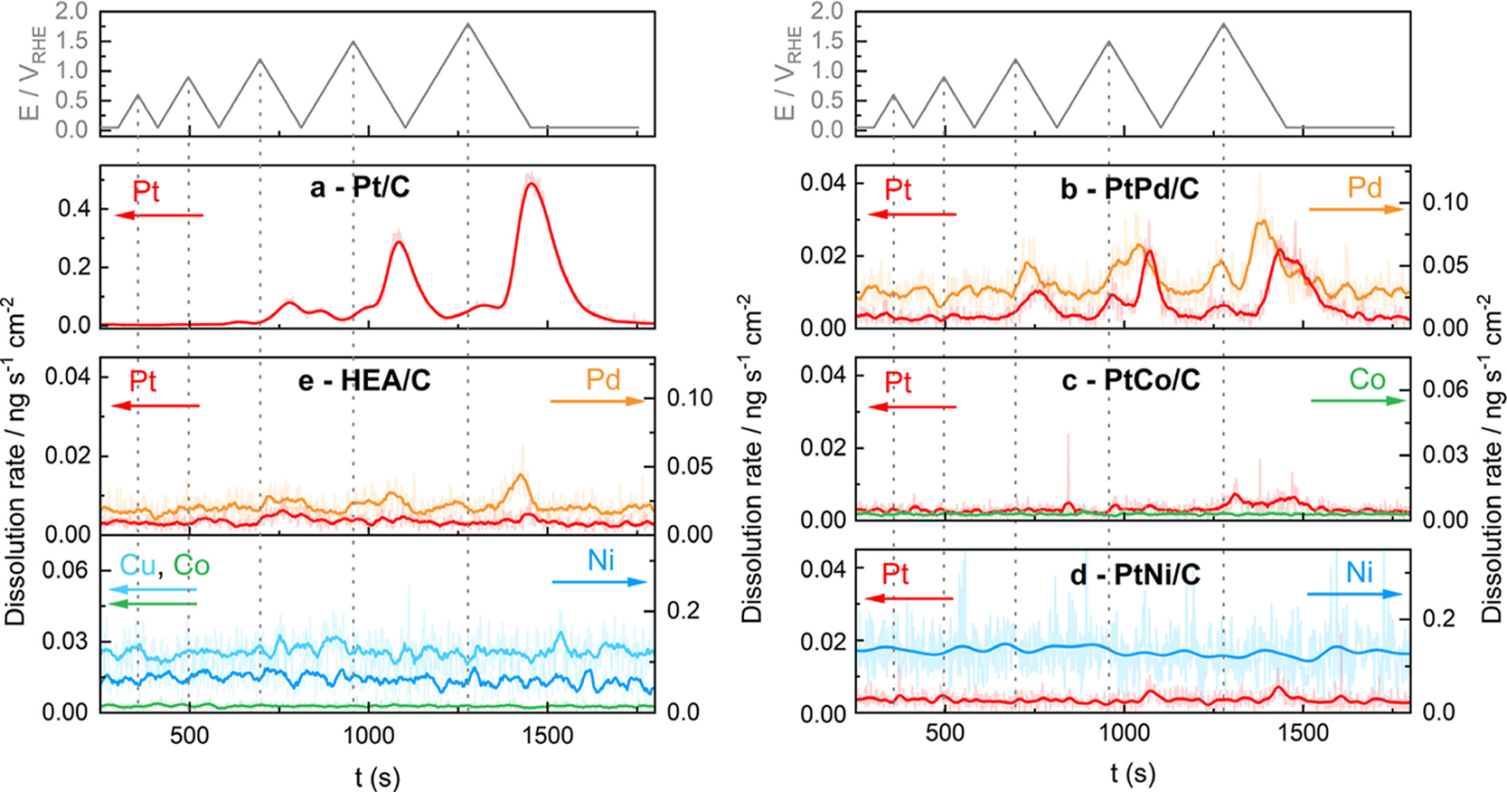
Stabilization effect of alloying on the dissolution of metals in 0.1 M KOH. Applied protocol (*Protocol A*) and the dissolution profiles of (a) Pt/C, (b) PtPd/C, (c) PtCo/C, (d) PtNi/C, and (e) PdCuPtNiCo/C (HEA/C). For clarity, the first 250 s (contact dissolution peaks) were excluded from this figure.

An obvious stabilization effect caused by alloying is observed. In contrast to Pt/C, no significant Pt dissolution is observed for PtPd/C during the second CV with the UPL of 0.9 *V*_RHE_ (Figure 5a,b). Anodic dissolution of Pt in PtPd/C occurs when the UPL is higher than 1.2 *V*_RHE_, and, interestingly, the anodic peaks are more clearly separated from the cathodic ones compared to Pt/C. Pd dissolution trends look similar to Pt, which is in agreement with the Pourbaix diagrams (Figures S7 and S8).^25^ Similar to Pt, transient dissolution due to the reduction of the formed oxidized species seems to prevail in the case of Pd. Cathodic dissolution of Pd, however, starts slightly earlier than that of Pt. Alloying Pt with other bimetallic alloys makes a difference. Non-noble metals have a strong stabilizing effect on Pt in alkaline media due to the stability of their oxides formed at anodic potentials in high-pH solutions. According to the Pourbaix diagrams in Figures S9 and S10, all the species formed at anodic potentials for Co and Ni are stable solid forms of (hydr)­oxides. Therefore, no transient dissolution due to the oxidation or reduction of Co and Ni species is expected, which stabilizes the surfaces of these alloys. Therefore, Pt dissolution in PtNi/C or PtCo/C is insignificant and becomes obvious only at the UPLs of 1.5 *V*_RHE_ and higher (Figure 5c,d).

Based on these results, one can say that alloying Pt with non-noble metals is beneficial for its stability. Figure 5e presents the dissolution profiles of Pt, Pd, Co, Ni, and Cu in PdCuPtNiCo/C in 0.1 M KOH. Pt dissolution in PdCuPtNiCo/C is significantly lower than in PtPd/C. The anodic and cathodic dissolution peaks are difficult to separate, as even the highest UPL used in this protocol (1.8 *V*_RHE_) is not high enough to trigger significant Pt dissolution. While Pd had a detrimental effect on structural stability in acidic pH, in the case of the alkaline pH, Pd was stabilized by the non-noble metals. Pd dissolution is observed but is much lower than in PtPd/C. Pd keeps the dissolution trends–all the anodic and cathodic dissolution peaks are present here, and it is still the least stable element in the alloy. According to Figures S16,S17, Pd has the most pronounced dissolution during the initial electrode/electrolyte contact. As non-noble metals are stable in a wide potential range at pH 13, they stabilize Pt and Pd in the alloy, as predicted. However, such stabilization can often come with a trade-off, and the dissolution of less stable elements can increase the dissolution of more stable ones by dragging their atoms from the surface of the materials during the dissolution.^26^ In the case of PdCuPtNiCo/C, the dissolution of Pt and Pd does not trigger additional Co, Ni, or Cu dissolution in alkaline electrolyte, except for Ni dissolution during contact (Figures S16,S17).

Figure S18 shows the dissolution profiles obtained for *Protocol B*. The trends of Pt dissolution are quite similar to those observed at low pH. Pt in the alloys exhibits no anodic dissolution except for the PtPd/C, where we can see both anodic and cathodic peaks. In other bimetallic alloys, only cathodic dissolution of low intensity can be seen. PdCuPtNiCo/C is the most stable alloy, which makes it useful for applications in fuel cells during start-up/shut-down operations. Moreover, PdCuPtNiCo/C obtains the highest current density among the tested alloys at the UPL of 1.8 *V*_RHE_ (Figure S19) while still demonstrating a lower dissolution of Pt and Pd and no dissolution of Ni, Co, and Cu. These findings suggest that the activity of this alloy is its intrinsic property and comes from the unique active sites rather than from the material’s dissolution, which highlights its higher stability. Moreover, PtPd/C also demonstrates high activity, suggesting that Pd might be the main contributor to the OER activity of the HEA/C. Notably, this HEA has previously shown high activity toward the ORR in alkaline media,^24,55–200^ in the potential range ca. 0.1–1.1 *V*_RHE_, where the alloy also demonstrates no detectable dissolution, underlining its promising application as an ORR catalyst in KOH.

The described stabilization effects can be seen better if we integrate the recorded dissolution profiles and compare the total and normalized dissolution in the alloys (Figure 6), as we did in the case of the acidic electrolyte.

**Figure 6:**
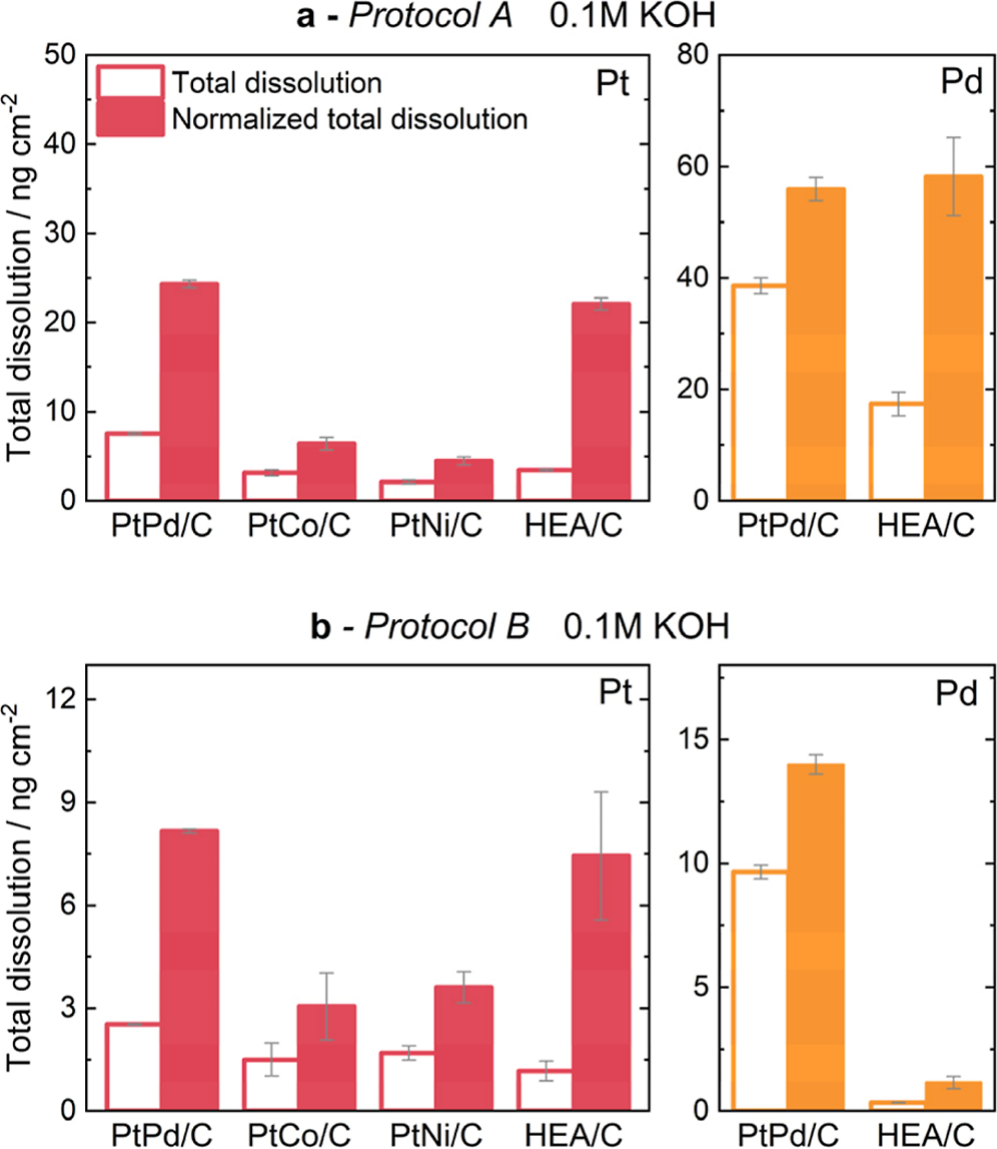
Total and normalized dissolution of the materials during (a) *Protocols A* and (b) *B* in alkaline electrolyte (without the contact peak). Cu, Ni, and Co dissolution is not presented for the alkaline media, as there is no detectable dissolution.

As the non-noble metals are stable at high pH and no detectable dissolution was observed in either protocol, we do not include them in the diagram in Figure 6. Similar to the results obtained for the acidic electrolyte, the alloys are less stable and dissolve more during the potentiodynamic protocol (*Protocol A*, Figure 6a) than during the potentiostatic holds (*Protocol B*, Figure 6b). Pt is still very stable in PtNi/C and PtCo/C, but in contrast with the acidic environment, Pt is also stable in PdCuPtNiCo/C. Of course, considering the normalized values changes the picture. Although the amount of dissolved Pt is the lowest in the PdCuPtNiCo/C, when normalized by the fraction of Pt in the alloy, Pt in PtNi/C and PtCo/C is more stable. This analysis gives quite different results for Pd. The presence of non-noble metals in PdCuPtNiCo/C stabilizes Pd in the structure and lowers its dissolution, especially during *Protocol B* (Figure 6b). Comparing the normalized total dissolution also shows that Pd is more stable in HEA NPs.

## Conclusion

Alloying has a significant impact on the stability of the electrocatalysts. Pt is stabilized in bimetallic alloys with Co and Ni, where Pt does not dissolve anodically and, in some cases (in PtCo/C and PtNi/C), even cathodically. Formation of Pt-rich shells around PtM (M = Co or Ni) cores also protects the non-noble metals from dissolution. In contrast, for Pt alloys containing Pd, the persistence of Pd in the shell during contact with an acidic electrolyte results in its further dissolution and triggers the dissolution of other metals that are highly unstable when exposed to acid (Ni and Co). Our estimation of the number of atoms removed from the surface during the initial electrode/electrolyte contact supports our hypothesis about the destabilizing role of Pd in PdCuPtNiCo/C at low pH. A different scenario takes place at a high pH. The PdCuPtNiCo/C catalyst is much more stable in alkaline media. Even though Pd is the least stable element, at high pH, it experiences stabilization due to the addition of the most stable Co, Ni, and Cu. Therefore, Pd dissolution from HEA/C decreases drastically compared with that of the PtPd/C alloy.

This study highlights the critical role of alloy composition and electrolyte environment in dictating stability, with Pt gaining stability when combined with Co or Ni and Pd having a destabilizing effect that can be mitigated at a high pH. Moreover, this study advocates for a knowledge-driven design when it comes to the synthesis of complex compounds for electrocatalysis. On the basis of the demonstrated results, a follow-up work on high-entropy alloys with other first–row transition metals (e.g., Fe) or other noble metals (e.g., Ir) instead of Pd would be a valuable next step. Detailed and systematic dissolution studies are of extreme importance for understanding the trends in such complex systems.

## Supplementary Material



## Notes

STEM-EDS images; XRD results; scheme of the NP conversion to HEA nanoparticles; Pourbaix diagrams of Pt, Pd, Co, Ni, and Cu; electrochemical measurements results; and dissolution profiles and analysis of Pt/C, PtPd/C, PtCo/C, PtNi/C, and PdCuPtNiCo/C under *Protocols A* and *B* (PDF)
