# Supplementary material for: Elemental Stability in Mixed Noble and Non-Noble Metal High Entropy Alloy Nanoparticle Electrocatalysts
Source: Chem Mater. 2026 May 18;38(11):5732–43. doi: 10.1021/acs.chemmater.6c00856 (PMC13255094; doi:10.1021/acs.chemmater.6c00856)
Supplement: Supplementary file 1 [file cm6c00856_si_001.pdf]

Supporting Information

Elemental Stability in Mixed Noble and Non-Noble Metal High Entropy Alloy Nanoparticle Electrocatalysts

*Tatiana Priamushko [a, †, *], Nabojit Kar [b, †], Pâmella S. Rodrigues [a, c], Nilotpal Kapuria [b], Sara E. Skrabalak [b, *], and Serhiy Cherevko [a, *]*

[a] Forschungszentrum Jülich GmbH, Helmholtz Institute Erlangen-Nürnberg for Renewable Energy (IET-2), Cauerstr. 1, 91058 Erlangen, Germany;

[b] Department of Chemistry, Indiana University Bloomington, Bloomington, Indiana 47405, United States;

[c] São Carlos Institute of Chemistry, University of São Paulo, Av. Trab. Sancarlense 400, 13566-590, São Carlos, Brazil;

[†] T.P. and N.K. contributed equally to this work

[*] Corresponding Authors: Tatiana Priamushko (tatyana.pryamushko@gmail.com), Sara E. Skrabalak (sskrabal@iu.edu), and Serhiy Cherevko (s.cherevko@fz-juelich.de).

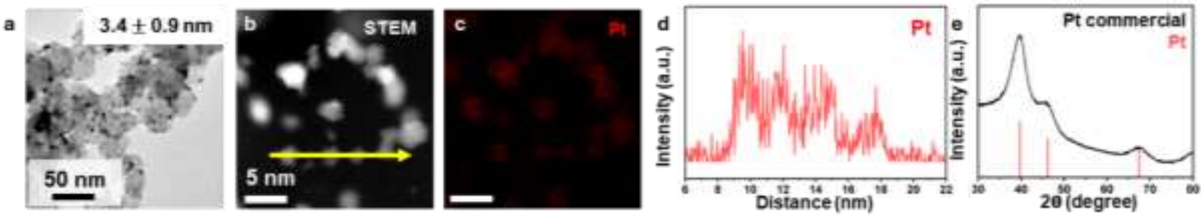

Figure S1. Characterization of commercial Pt NPs. a) TEM image, b) STEM image and c) STEM-EDS elemental mapping where red indicates Pt, d) linescan analysis according to yellow line in b), and e) XRD pattern. Reference: Pt (ICSD: 243678).

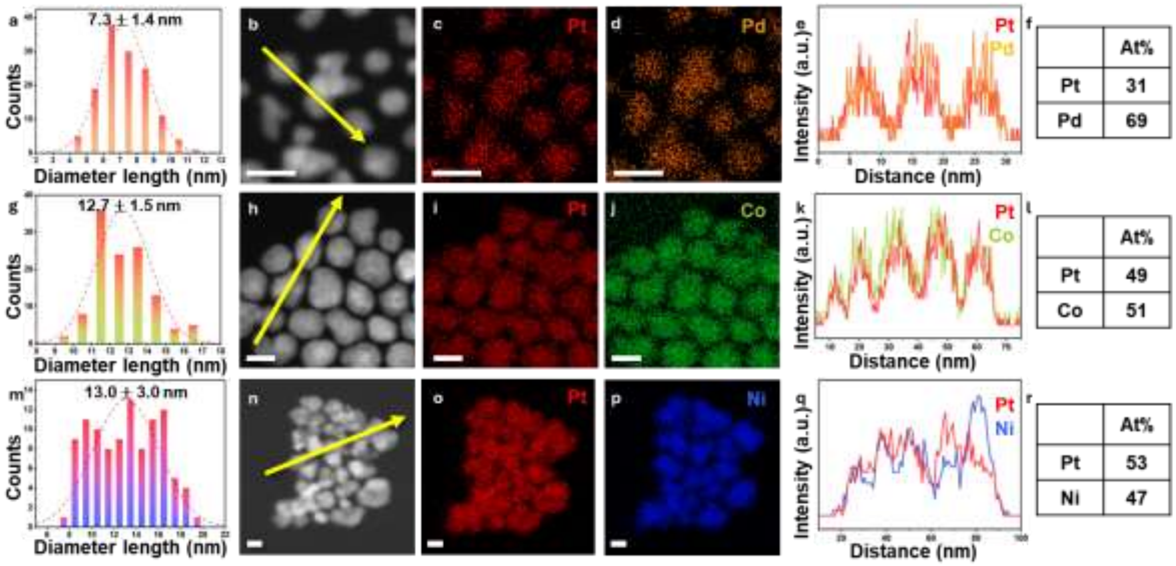

Figure S2. Characterization of Pt-containing bimetallic NPs. TEM images, STEM-EDS elemental mapping, linescan analysis according to yellow lines, and table showing the atomic percentages taken by SEM-EDS of the samples (a-f) PtPd, (g-l) PtCo, and (m-r) PtNi NPs.

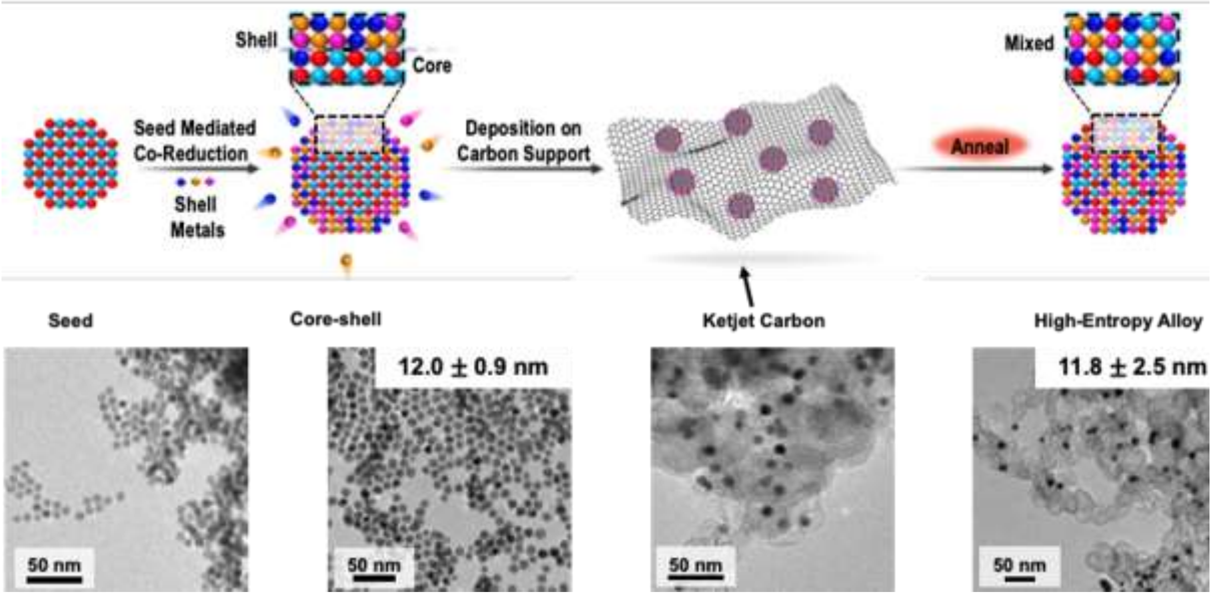

Figure S3. (top) Scheme of the NP conversion strategy to HEA NPs, where bimetallic seeds are first prepared and a trimetallic shell is deposited through seed-mediated co-reduction to prepare core-shell NPs. These NPs are then supported on carbon and annealed to facilitate intermixing and the formation of the HEA NPs. (bottom) TEM images of the product NPs at each step in the synthesis of PdCuPtNiCo NPs supported on carbon.

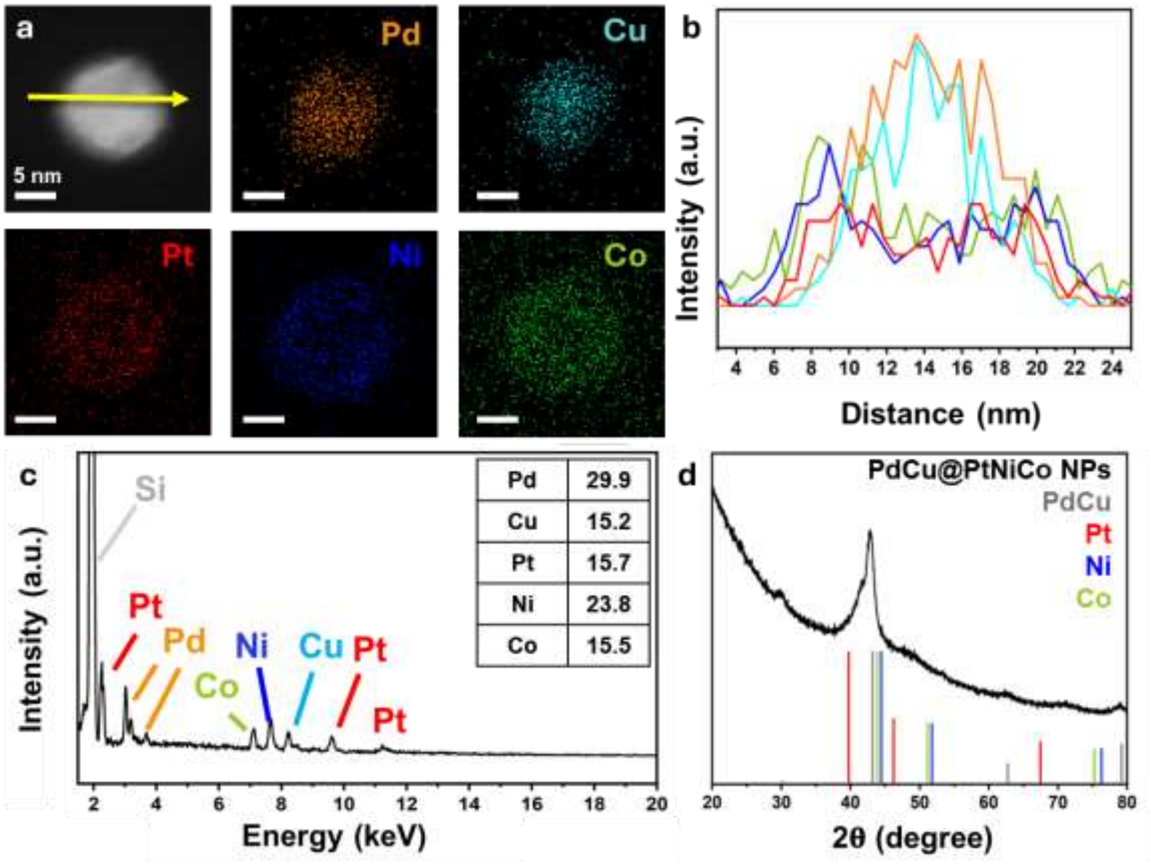

Figure S4. Characterization of core-shell PdCu@PtNiCo NPs. (a) STEM-EDS elemental mapping, (b) linescan analysis according to yellow lines, (c) SEM-EDS spectrum of an ensemble of NPs. Si signal is from the Si wafer on which the NPs were dropcasted. Inset table showing the atomic percentages taken by SEM-EDS of the samples, and (d) XRD pattern. References: PdCu (ICSD: 166154), Pt (ICSD: 243678), Ni (ICSD: 260172), and Co (ICSD: 52934)

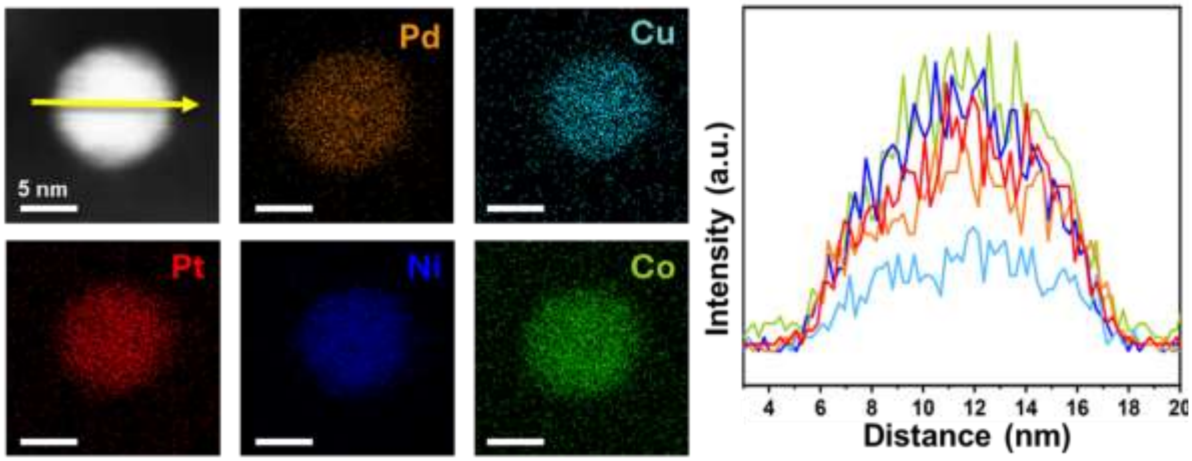

Figure S5. STEM-EDS elemental mapping and linescan analysis according to yellow lines of PdCuPtNiCo HEA NPs.

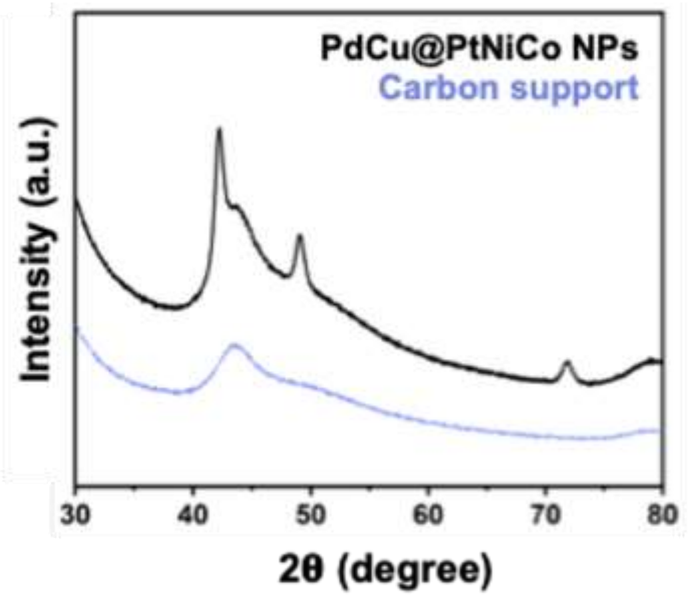

Figure S6. XRD pattern of PdCuPtNiCo/C HEA with reference with ketjen carbon support.

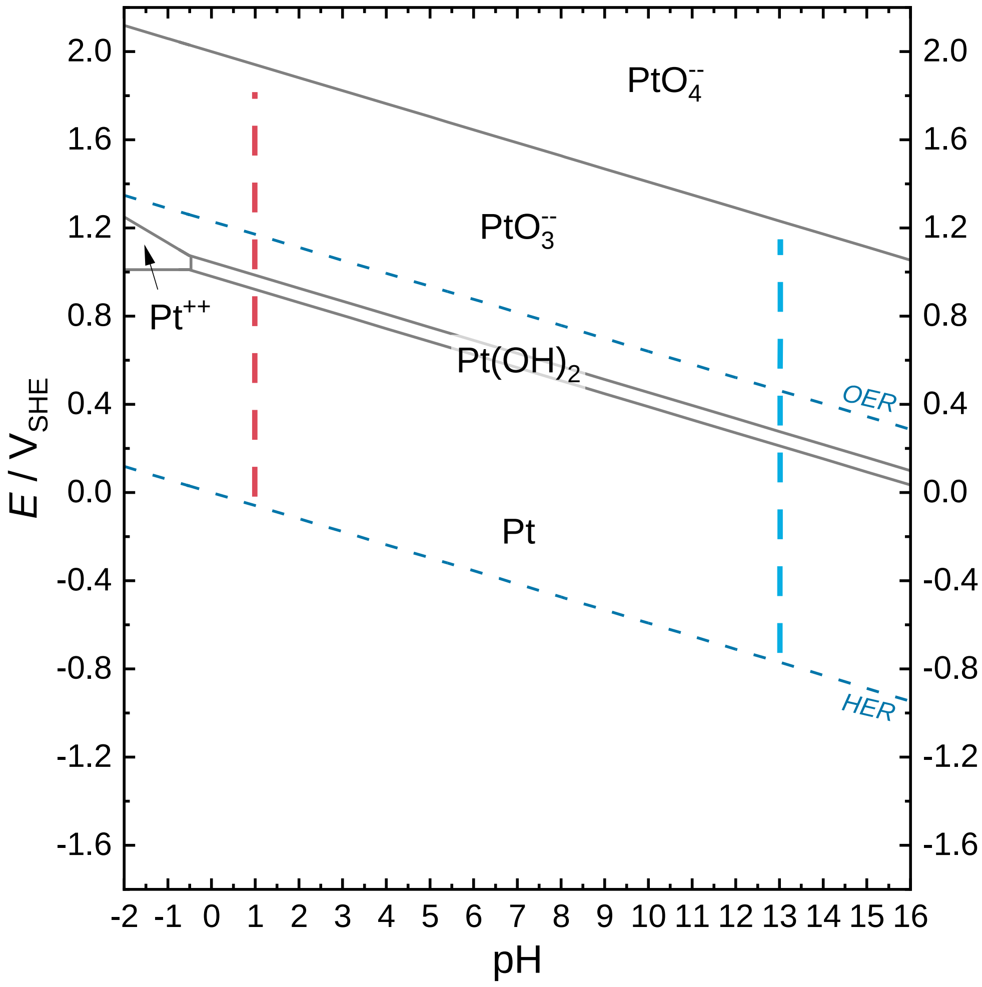

Figure S7. Pourbaix diagrams of platinum calculated from experimental thermodynamic tables[1] with aqueous ion concentration of 10^{-6} M at 25 °C. The red and blue areas indicate the potential range at low and high pH values at which all the experiments were performed in this work, respectively.

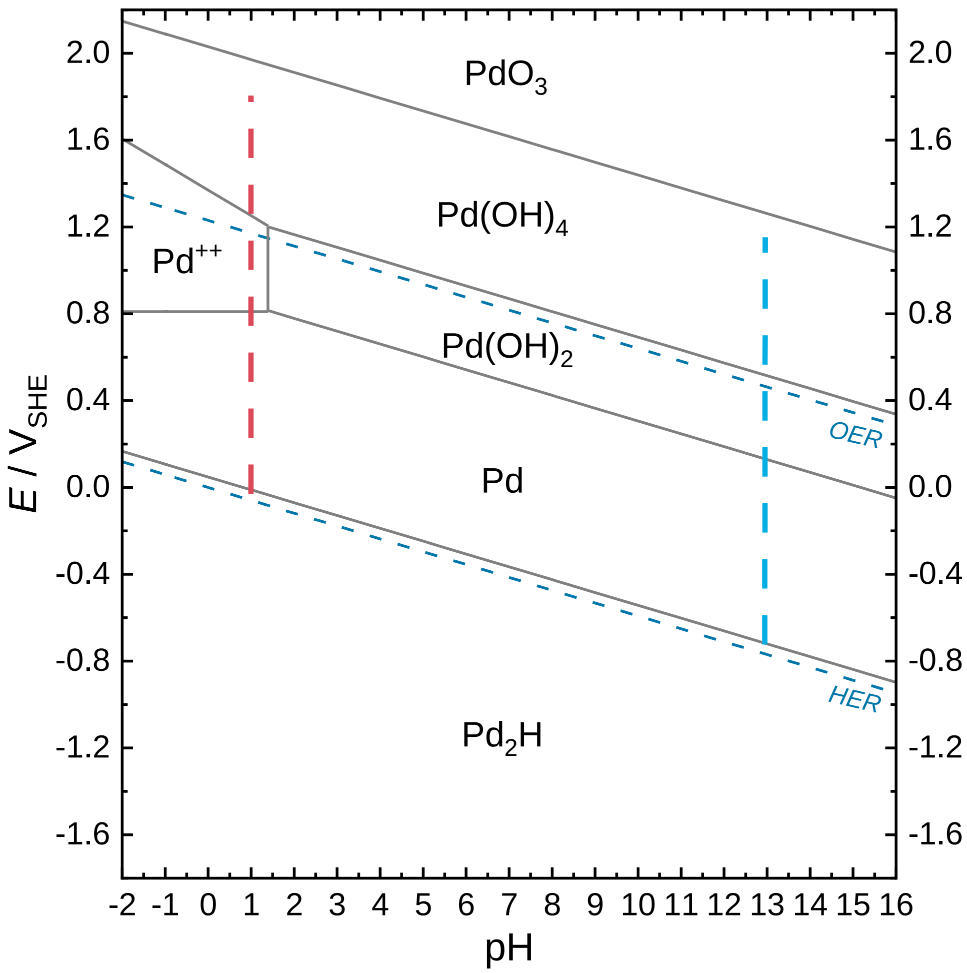

Figure S8. Pourbaix diagrams of palladium calculated from experimental thermodynamic tables[1] with aqueous ion concentration of 10^{-6} M at 25 °C. The red and blue areas indicate the potential range at low and high pH values at which all the experiments were performed in this work, respectively.

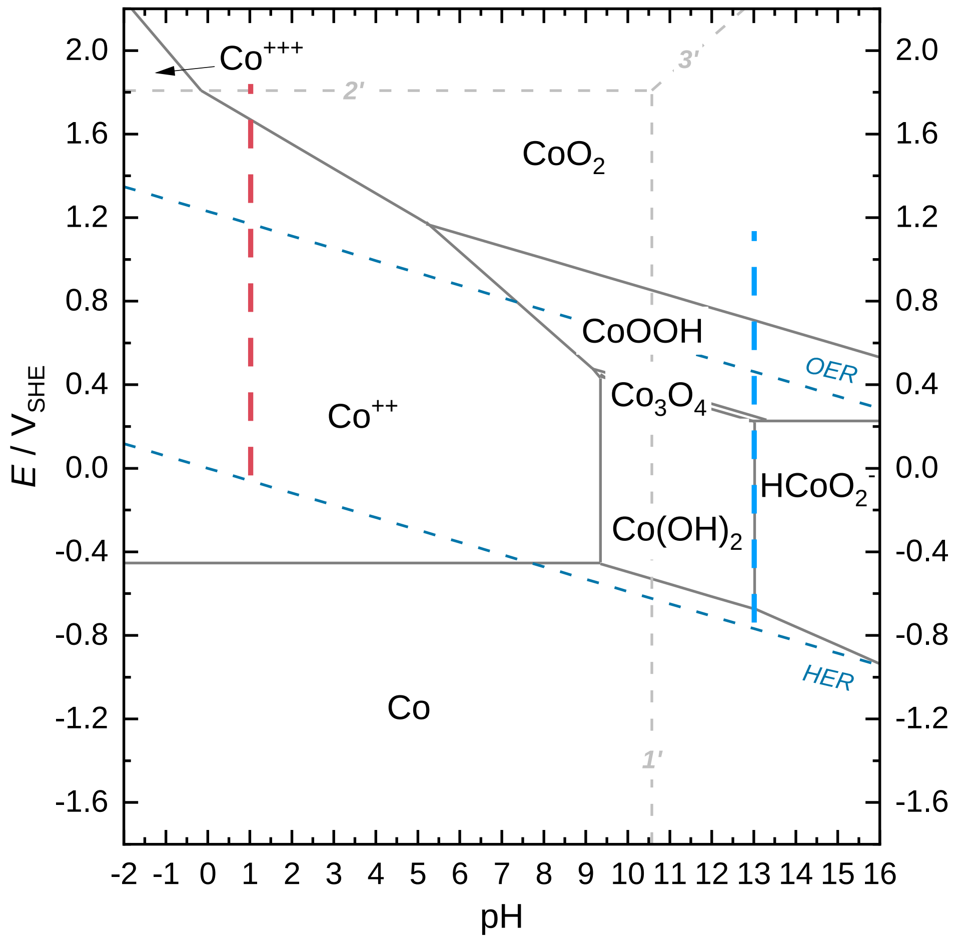

Figure S9. Pourbaix diagrams of cobalt calculated from experimental thermodynamic tables[1] with aqueous ion concentration of 10^{-6} M at 25 °C. The red and blue areas indicate the potential range at low and high pH values at which all the experiments were performed in this work, respectively. The faint dashed lines represent the limits of the domains of the relative predominance of the dissolved substances according to the following data:

1′: Co^{++} / $HCoO^{-}{}_{2}$ *pH=10.57*

2′: Co^{2+} / Co^{3+} *E_0=1.808*

3′: $HCoO^{-}{}_{2}$ / Co^{3+} *E_0=-0.065+0.1773pH*

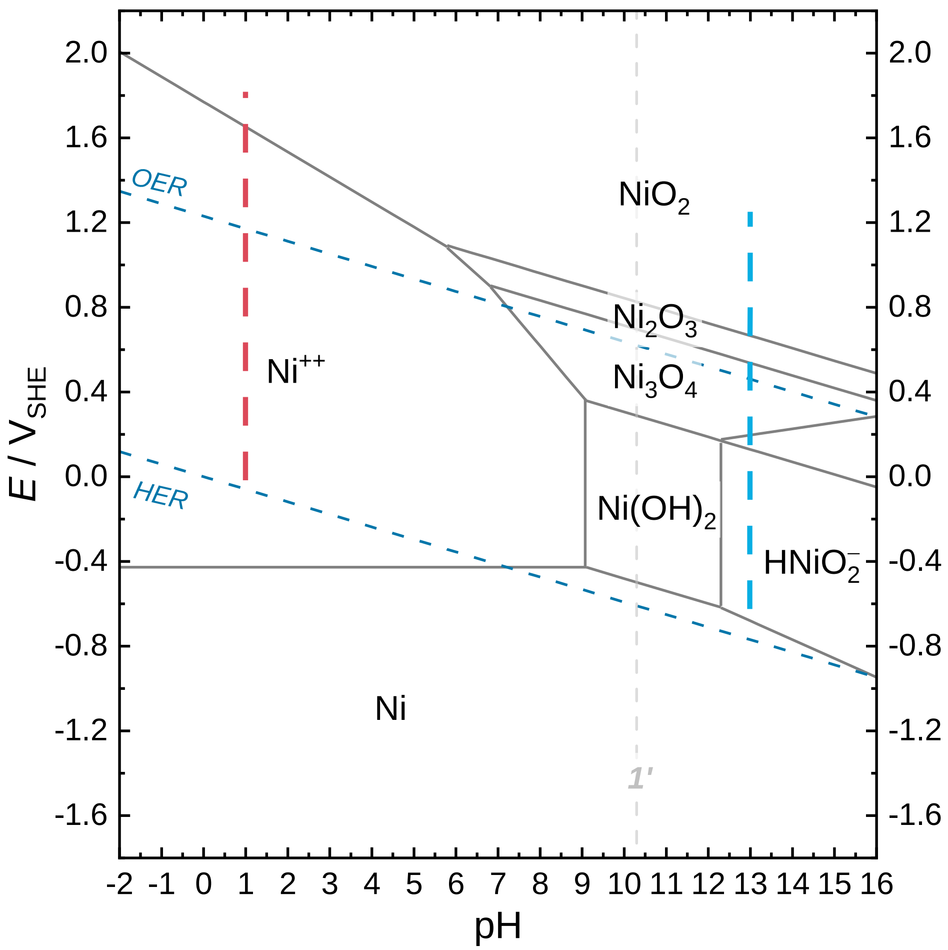

Figure S10. Pourbaix diagrams of nickel calculated from experimental thermodynamic tables[1] with aqueous ion concentration of 10^{-6} M at 25 °C. The red and blue areas indicate the potential range at low and high pH values at which all the experiments were performed in this work, respectively. The faint dashed line represents the limits of the domains of the relative predominance of the dissolved substances according to the following data:

1′: Ni^{++} / $HNiO^{-}_{2}$ pH=10.13

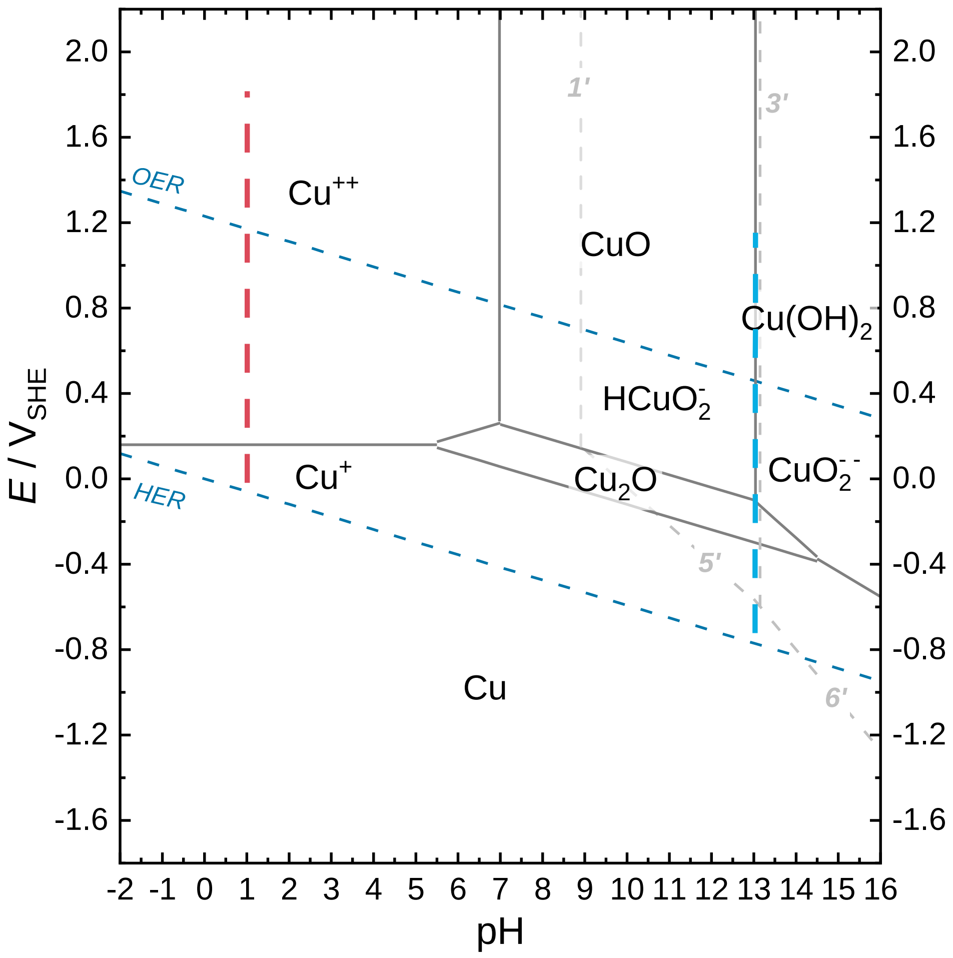

Figure S11. Pourbaix diagrams of copper calculated from experimental thermodynamic tables[1] with aqueous ion concentration of 10^{-6} M at 25 °C. The red and blue areas indicate the potential range at low and high pH values at which all the experiments were performed in this work, respectively. The faint dashed lines represent the limits of the domains of the relative predominance of the dissolved substances according to the following data:

1´: $Cu^{++} / HCuO^{-}_{2}$ pH=8.91

2´: Cu^{++} / CuO^{--}_{2} pH=9.97

3´: $HCuO^{-}_{2} / CuO^{--}_{2}$ pH=13.15

4´: Cu^{+} / Cu^{++} E_0=0.153

5´: $Cu^{+} / HCuO^{-}_{2}$ E_0=1.733 – 0.1773pH

6´: Cu^{+} / CuO^{--}_{2} E_0=2.510 – 0.2364pH

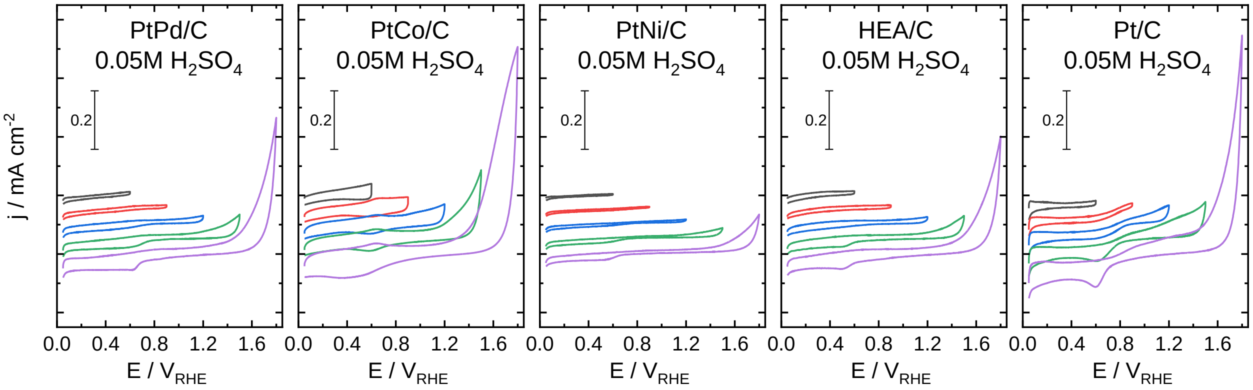

Figure S12. Cyclic voltammetry (CV) of the alloys was recorded during *Protocol A* in 0.05M H_2SO_4. Each following CV is shifted lower to avoid overlapping of the data for the sake of clarity.

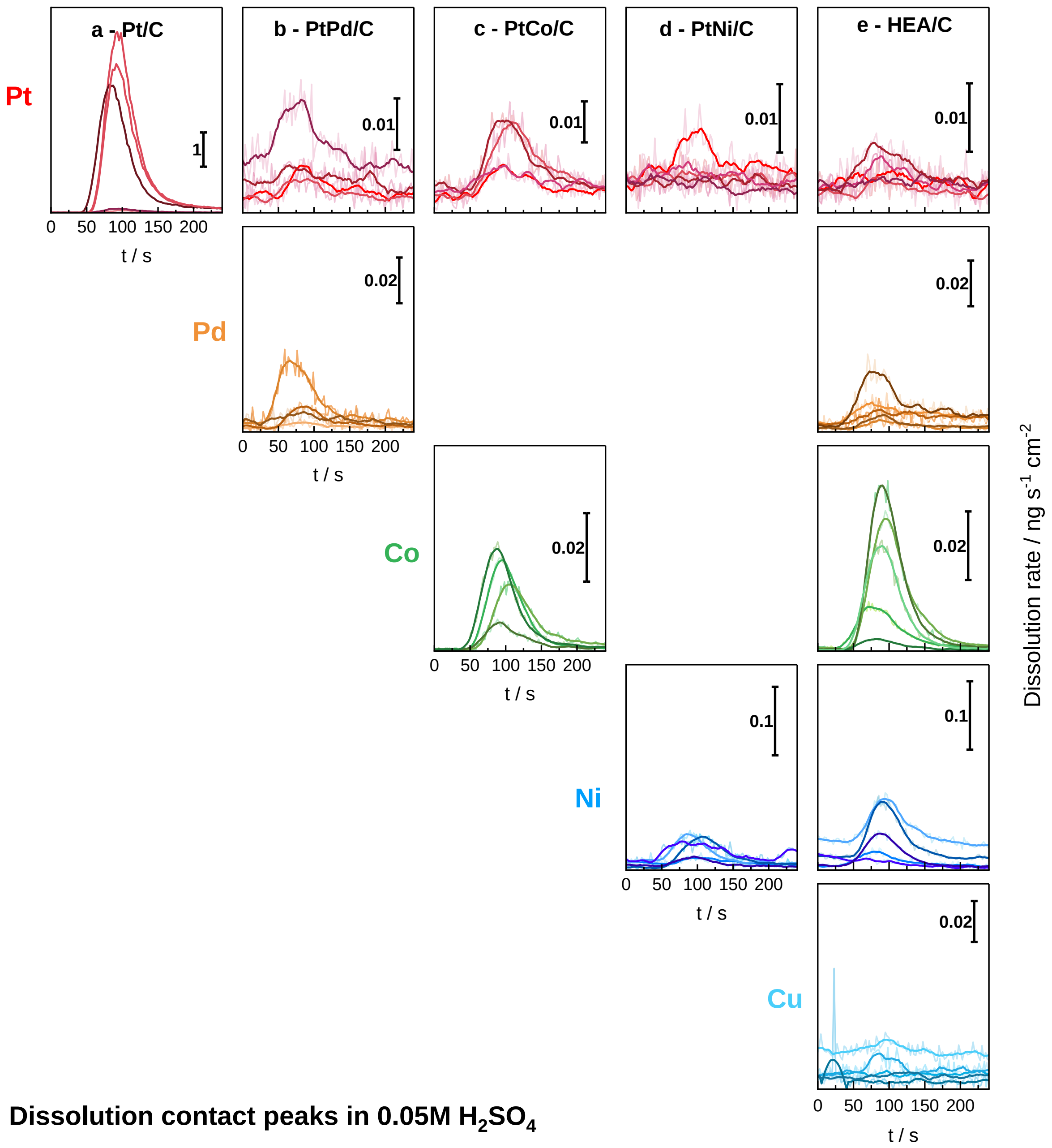

Figure S13. Dissolution profiles during the electrode/electrolyte initial contact (contact peak) in 0.05M H_2SO_4 for (a) Pt/C, (b) PtPd/C, (c) PtCo/C, (d) PtNi/C, and (e) HEA/C. The graph depicts the contact peaks registered in both *Protocol A* and *Protocol B* to show the reproducibility.

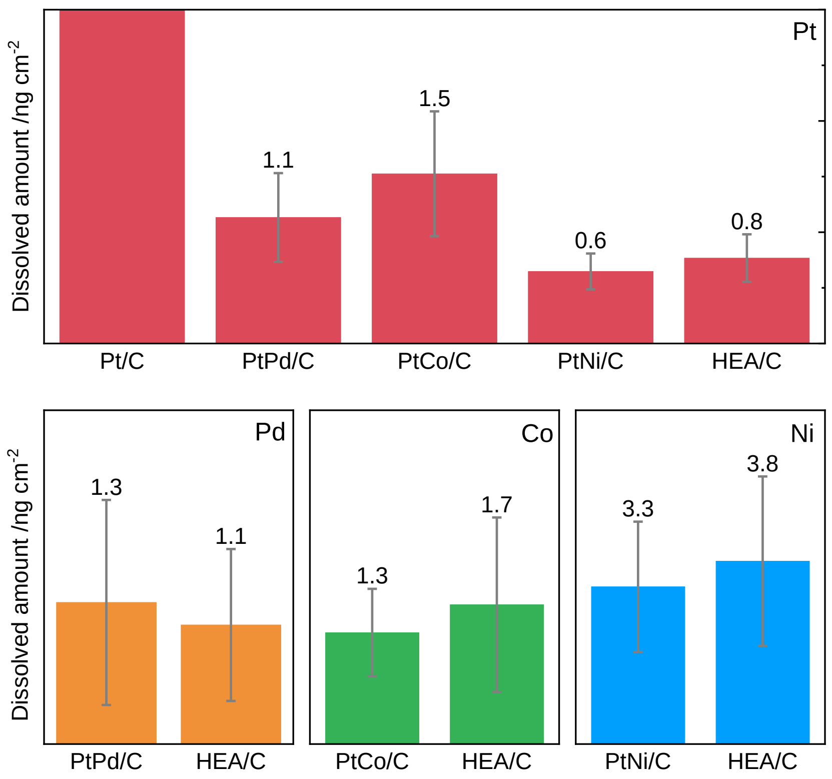

Figure S14. Comparison of the dissolution due to the electrode/electrolyte initial contact (contact peak) in 0.05M H_2SO_4 for Pt, Pd, Co, and Ni in Pt/C, PtPd/C, PtCo/C, PtNi/C, and HEA/C alloys. The diagram is obtained by integrating the dissolution profiles depicted in Figure S13. The dissolution of Cu in HEA/C was estimated to be 1.3±0.4 ng cm^{-2}.

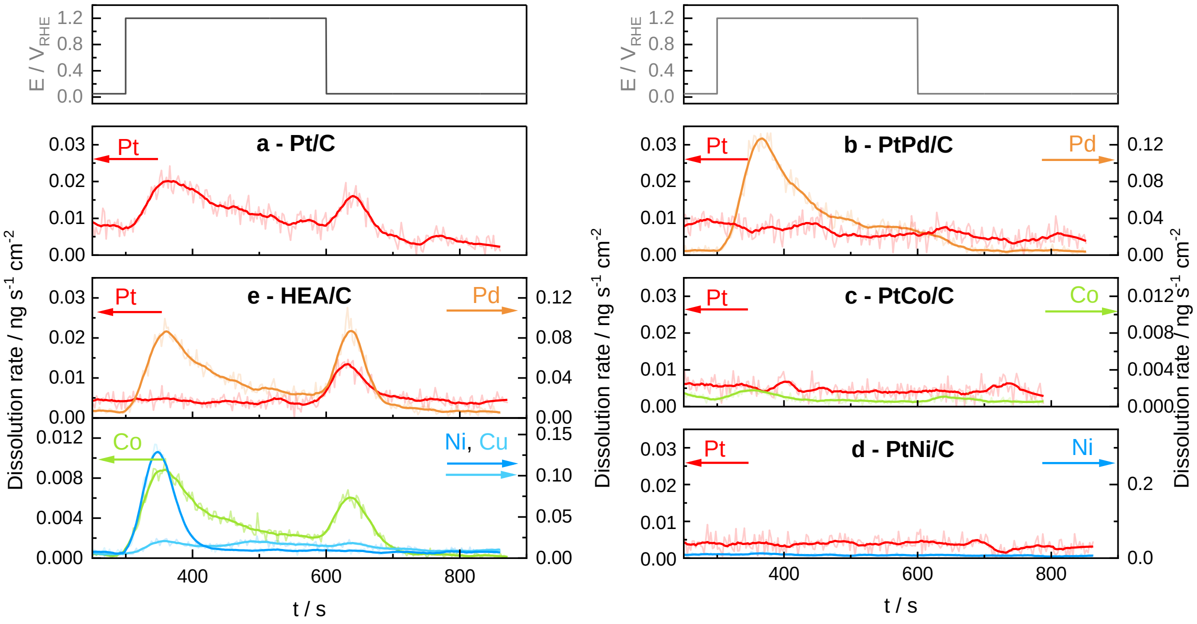

Figure S15. The stabilization effect of the alloying on the durability of metals in 0.05M H_2SO_4. Applied protocol (*Protocol B*) and the dissolution profiles (a) Pt/C, (b) PtPd/C, (c) PtCo/C, (d) PtNi/C, and (e) PdCuPtNiCo/C (HEA/C). For clarity, the first 250 seconds (contact dissolution peaks) were excluded from this figure.

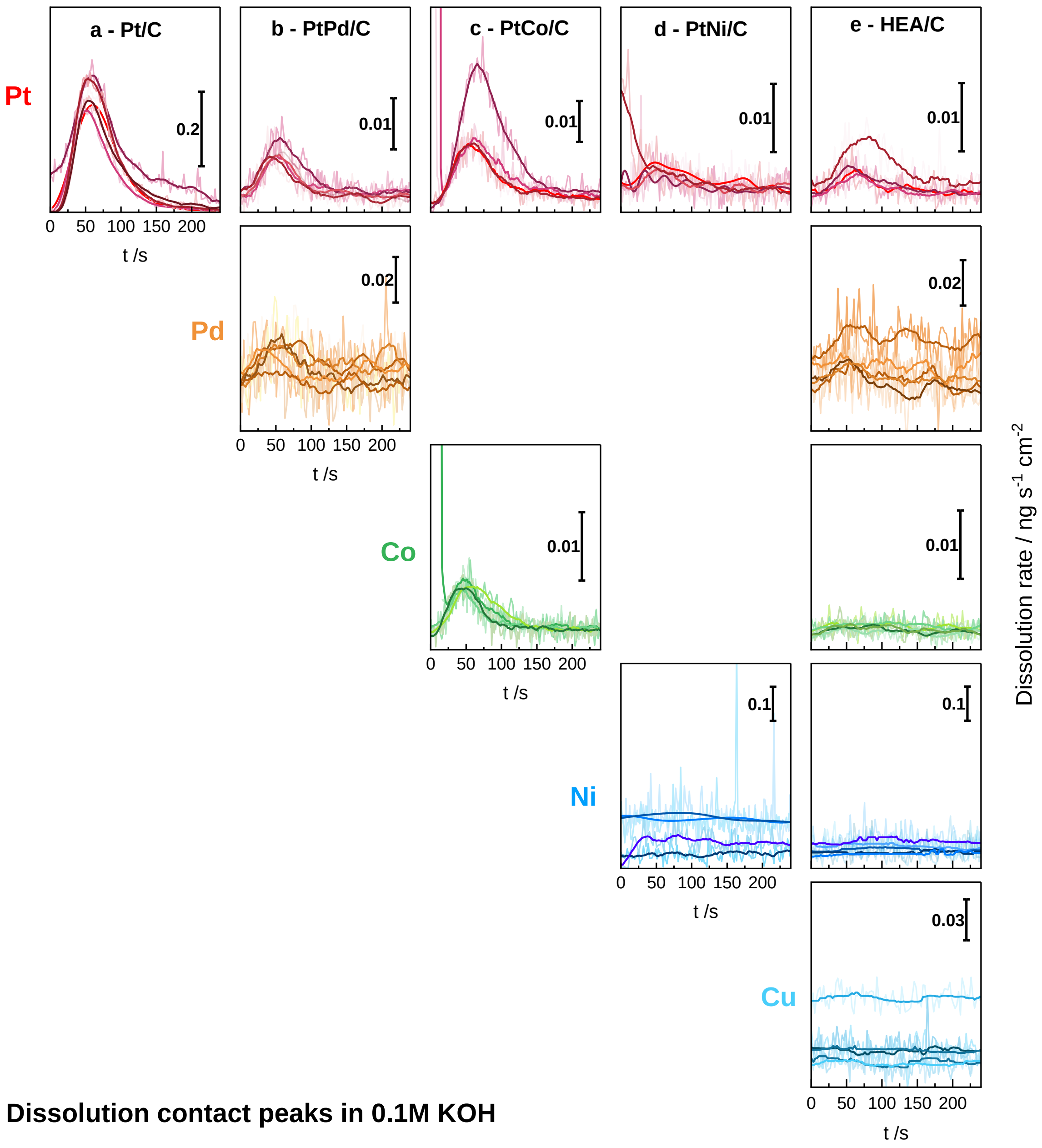

Figure S16. Dissolution due to the electrode/electrolyte initial contact (contact peak) in 0.1M KOH for (a) Pt/C, (b) PtPd/C, (c) PtCo/C, (d) PtNi/C, and (e) HEA/C. The graph depicts the contact peaks registered in both *Protocol A* and *Protocol B* to show the reproducibility.

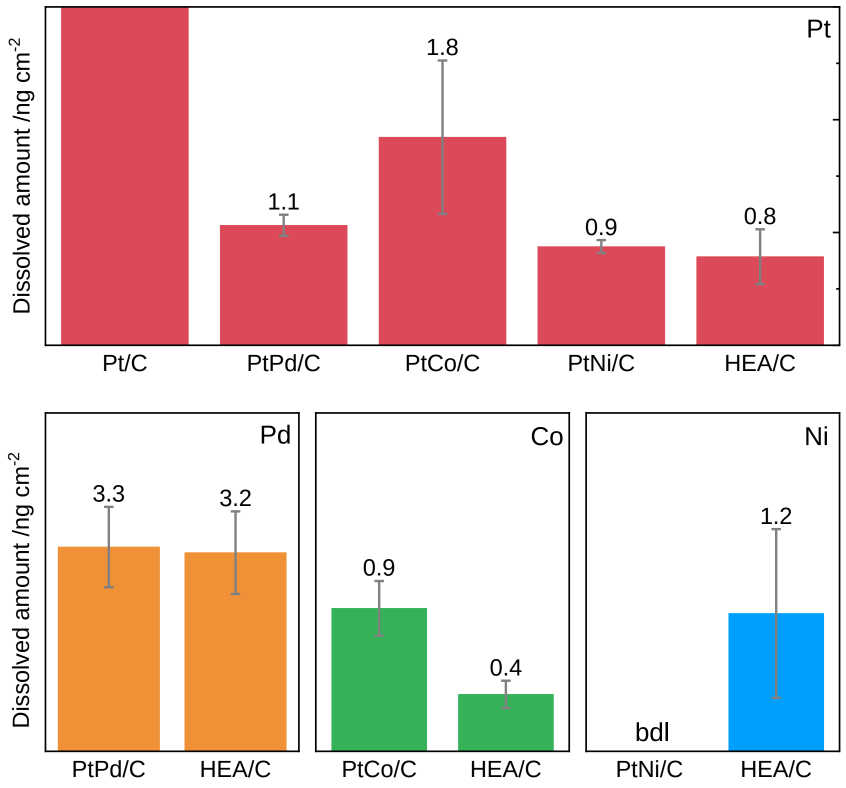

Figure S17. Comparison of the dissolution due to the electrode/electrolyte initial contact (contact peak) in 0.1M KOH for Pt, Pd, Co, and Ni in Pt/C, PtPd/C, PtCo/C, PtNi/C, and HEA/C alloys. The diagram is obtained by integrating the dissolution profiles depicted in Figure S16. The dissolution of Cu in HEA/C was also below the detection limit (bdl).

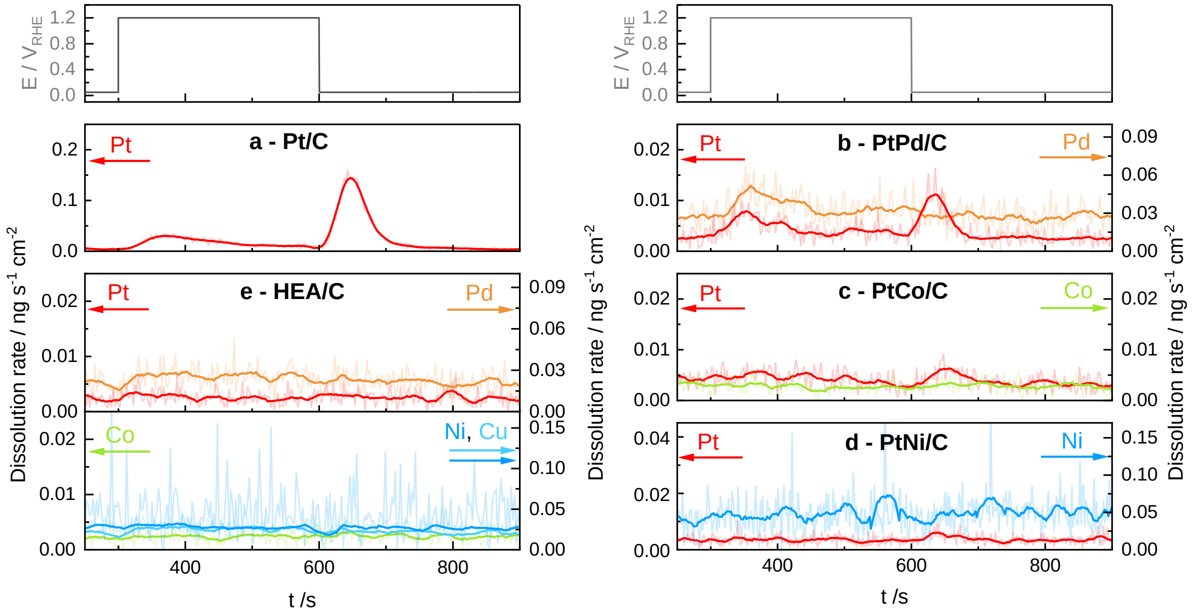

Figure S18. The stabilization effect of the alloying on the durability of metals in 0.1M KOH. Applied protocol (*Protocol B*), recorded current densities, and the dissolution profiles of (a) PtPd/C, (b) PtCo/C, (c) PtNi/C, (d) Pt/C, and (e) PtPdCuNiCo/C (HEA/C).

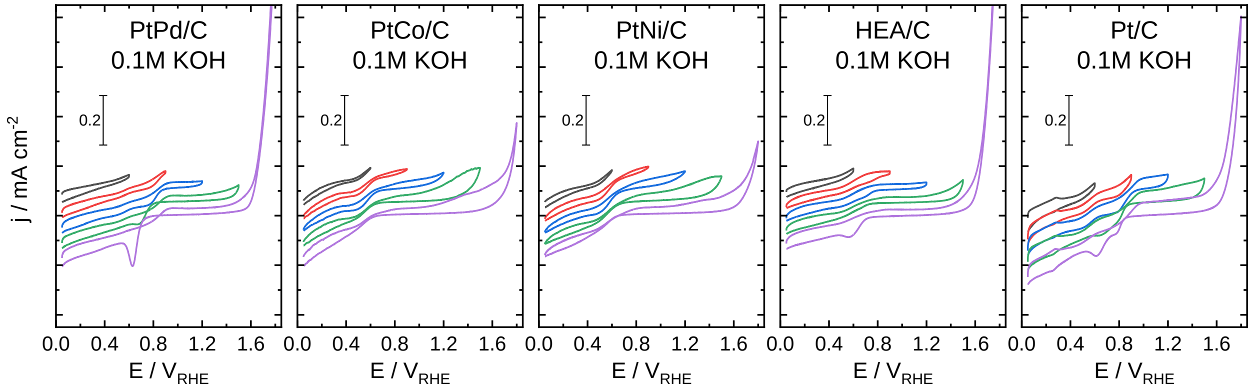

Figure S19. Cyclic voltammetry (CV) of the alloys was recorded during *Protocol A* in 0.1M KOH. Each following CV is shifted lower to avoid overlapping of the data for the sake of clarity.

Table S1. Composition, particle size, and theoretically estimated number of surface atoms for each constituent metal and non-metal, and the estimated number of atoms removed from the surface of the NPs during the measurements in H_2SO_4. Average size of the spot and of the dissolution were taken for the calculations.

Nanoparticle composition	Size (nm)	d (nm)	Theoretically estimated surface atom amount (from the geometry and the crystal structure)	Theoretically estimated surface atom amount (from the dissolved amounts)
$Pd_{0.3}Cu_{0.15}Pt_{0.16}Ni_{0.24}Co_{0.15}$	5.9	0.214	Pd = 8.54E+11	Pd = 1.31E+11
			Cu = 4.27E+11	Cu = 2.59E+11
			Pt = 4.55E+11	Pt = 5.19E+10
			Ni= 6.83E+11	Ni= 8.19E+11
			Co= 4.27E+11	Co= 3.65E+11
$Pt_{0.53}Ni_{0.47}$	6.5	0.216	Pt = 1.02E+12	Pt = 3.89E+10
			Ni = 9.04E+11	Ni = 7.11E+11
$Pt_{0.5}Co_{0.5}$	6.3	0.218	Pt = 1.03E+12	Pt = 9.72E+10
			Co = 1.03E+12	Co = 2.79E+11
$Pd_{0.7}Pt_{0.3}$	3.6	0.226	Pd = 2.29E+12	Pd = 1.54E+11
			Pt = 1.03E+12	Pt = 7.13E+10

Estimation of Surface Atoms of the Nanoparticles

Surface atoms of alloy nanoparticles were estimated assuming spherical particles and an FCC crystal structure. The particle radius (r) was obtained from TEM/XRD analysis and the lattice parameter (a) was calculated from the XRD d-spacing. The following equations were used.

Equations

Eq. 1 $A\_NP = 4\pi r^2$ (Surface area of a nanoparticle)

Eq. 2 $a = \sqrt{3}\ d$ (Lattice parameter from XRD spacing)

Eq. 3 $A\_cell = a^2$ (Surface area of one FCC surface unit cell)

Eq. 4 $N\_cell{,}surface = A\_NP / A\_cell$ (Number of surface unit cells)

Eq. 5 $N\_surface{,}NP = (A\_NP / a^2) \times 2$ (Surface atoms per nanoparticle; 2 atoms per FCC surface cell)

Eq. 6 $V\_NP = (4/3)\pi r^3$ (Volume of a nanoparticle)

Eq. 7 $N\_cell{,}volume = V\_NP / a^3$ (Number of FCC unit cells in nanoparticle volume)

Eq. 8 $N\_total{,}NP = (V\_NP / a^3) \times 4$ (Total atoms per nanoparticle; 4 atoms per FCC unit cell)

Eq. 9 Total mass of nanoparticles deposited

$$m_{NP} = L \times A_{spot} \times f$$

where

L = catalyst loading (g cm^{-2}) for this paper ~2.5 ug/cm^2

A_{spot} = electrode spot area (cm^2) for this paper 0.021 cm^2

f = metal loading fraction (for this paper 10 wt%)

Eq. 10 Total number of atoms in the nanoparticle loading

$$N_{total} = \frac{m_{NP}}{M} \times N_A$$

where
M = molar mass of the alloy
N_A = Avogadro's number.

Eq. 11 Total number of surface atoms in the catalyst loading

$$N\_surface, total = N\_total \times ((N\_surface, NP)/(N\_surface, NP + N\_cell, volume))$$

Example Parameters Used in This Work

Parameter	Value
Nanoparticle radius (r)	6.5 nm
XRD d-spacing (d)	0.2165 nm
Calculated lattice parameter (a)	0.375 nm
Alloy composition	$Pt_{53}Ni_{47}$
Alloy molar mass	130.98 g mol^{-1}
Catalyst loading	2.5 μg cm^{-2}
Electrode spot area	0.021 cm^2
Metal fraction	0.1

Using these parameters, the number of surface atoms and total atoms for the nanoparticle loading were calculated following the equation chain above.

Eq. 12 The calculations of estimated number of atoms removed from the surface of the NPs during the measurements:

$$m_{contact} = DA_{contact} * S_{spot},$$

where $m_{contact}$ is a mass of the catalyst removed from the spot during the initial contact between the electrode and electrolyte, $DA_{contact}$ – dissolved amounts of the alloy removed during the initial contact between the electrode and electrolyte (values presented in Figure S14), S_{spot} – the average area of the spot.

Eq. 13 $N_{dissolved} = \frac{m_{contact}}{m_{atom}}$,

Where $N_{dissolved}$ is the number of atoms removed from the surface and m_{atom} is the mass of one atom.

References

(1) POURBAIX, M. Atlas of Electrochemical Equilibria in Aqueous Solutions. *NACE* **1966**.
